# Emphasizing the Communal Demands of a Leader Role Makes Job Interviews Less Stressful for Women But Not More Successful

**DOI:** 10.1007/s11199-024-01509-7

**Published:** 2024-10-28

**Authors:** Christa Nater, Alice H. Eagly, Madeline E. Heilman, Nadine Messerli-Bürgy, Sabine Sczesny

**Affiliations:** 1 Department of Psychology, University of Bern, Bern, Switzerland; 2 Department of Psychology, Northwestern University, Evanston, IL USA; 3 Department of Psychology, New York University, New York, USA; 4 FADO, Institute of Psychology, University of Lausanne, Lausanne, Switzerland

**Keywords:** Agency and communion, Gender, Job interview, Leadership, Role incongruity, Salivary cortisol

## Abstract

The cultural construal of leadership as masculine impedes women’s attainment of leader roles. This research examined whether adding feminine demands to a leader role relieved the greater stress experienced by women than men in a job interview for a leadership position and considered the processes that mediated women’s less favourable interview outcomes. In a hiring simulation, management students (*N* = 209; 112 women, 97 men) interviewed for a leader role framed by either stereotypically feminine or masculine role requirements. As shown by the stress biomarker salivary cortisol, the feminine role framing alleviated women’s, but not men’s, physiological stress response during the interview. However, under both masculine and feminine role framing, women, compared with men, reported lesser fit, expected poorer interview performance, appraised greater threat relative to challenge, and evaluated their performance less favourably, as did external raters. An additional vignette study (*N* = 305; 189 women, 111 men, 5 diverse) found that the feminine role framing increased the leader role’s communal demands but still conveyed strong agentic demands not different from those of the masculine role. In conclusion, although a feminine role framing alleviated women’s physiological stress response, it did not change their less favourable outcomes, as indicated by participants’ self-reports and others’ reports.

**Supplementary Information:**

The online version contains supplementary material available at 10.1007/s11199-024-01509-7.

Far more men than women hold the most influential and high-status leadership positions within organizational hierarchies (World Economic Forum, 2024), consistent with the cultural construal of leadership as masculine (Koenig et al., 2011). This underrepresentation of women in leadership is likely determined by multiple factors (see review by Lyness & Grotto, 2018). Explanations range from women’s lesser interest and aspirations (Netchaeva et al., 2022) to role incongruity arising from the mismatch between leadership stereotypes and the female gender stereotype (role incongruity theory; Eagly & Karau, 2002; lack of fit model; Heilman, 1983; Heilman et al., 2024). It is this incongruity approach that we pursue in this research.

Role incongruity for female leaders arises because the stereotypical belief that women have predominantly communal qualities (e.g., affectionate, caring) is incongruent with the primarily agentic qualities that people believe are essential for leaders and managers (e.g., taking charge and directing others; Eagly et al., 2020; Koenig et al., 2011). For men, however, the demands of such leader roles coincide with the agentic qualities that are commonly ascribed to them (e.g., assertive, dominant). The resulting perception of incongruity induces negatively biased evaluations of women as potential and actual leaders (e.g., Carli, 2018; Heilman, 2012). In fact, both controlled experiments and audit studies have documented that people evaluate women less favourably for male-dominated (but not female-dominated) positions, even when the female candidates were described as identical to the male candidates in all aspects other than their gender (see meta-analyses by Eagly et al., 1992; Koch et al., 2015).

## Do Gender-Incongruent Leader Roles Affect Women’s Experiences in Job Interviews?

As illustrated by these findings, role incongruity and lack-of-fit research has predominantly focused on the observer perspective and has documented that observers judge women’s performances of leader positions as inferior to those of men. Role incongruity and lack-of-fit theories further indicate that women themselves can experience more difficulty as candidates for leader roles (Eagly & Karau, 2002; Heilman, 1983, 2012). With the goal to better understand women’s (and men’s) experiences in job interviews for leader roles, our research shifts the focus to self-perception.

Relevant to individuals’ lack-of-fit experiences for leadership, women on average accord themselves less agency and more communion than men (Hsu et al., 2021). As a result of their lower levels of self-ascribed agency on average, women experience greater lack of fit for leadership roles. In fact, vignette studies have shown that among management students, women compared to men reported lower self-ascribed fit for a leader role because they viewed themselves as less agentic (Bosak & Sczesny, 2008; Nater & Sczesny, 2016). Greater lack of fit for leadership roles has been theorized to lower women’s expectations for success and their evaluation of their own performance in these roles (see Heilman, 1983, 2012). Specifically, experiences of inferior or superior fit for leader roles “create a predisposition, or a cognitive set, toward negativity or positivity that colors judgment of self” (Heilman, 1983, p. 279). The present research provides an empirical test of this proposed link in the context of job interviews, as job interviews are a crucial moment in people’s striving for leadership. We examined whether women’s compared to men’s own lack-of-fit perceptions fostered more negative expectations for their performance in interviews, which in turn yield less favourable self-evaluations of one’s performance.

This study used simulated job interviews for a leader role with live interaction to investigate the consequences of lack-of-fit perceptions that affect women more than men who interview for leadership. With its in-person methodology, our study advances existing role incongruity research beyond the more typical vignette studies. In fact, the present research advances incongruity theories by assessing both physiological stress responses as well as self-reported threat versus challenge appraisal after the job interview in an ecologically valid in-person setting. Our work thus meets the criticism that research on gender and leadership has relied too exclusively on self-report measures (Peterson & Bartels, 2017).

## Gender-Incongruent Leader Roles May Stress Women in Job Interviews

Women’s perceived lack of fit for leadership and the resulting negative performance expectations likely elicit physiological stress because they set up conditions for being worried (i.e., threat) rather than eager (i.e., challenge). Specifically, as posited by the biopsychosocial model of challenge and threat (Blascovich & Tomaka, 1996; Seery, 2013), physiological stress responses reflect the ratio between a person’s appraisal of the task demands and perceived resources to cope with them, with threat following from demands exceeding resources and challenge from sufficient coping resources. In a job interview for a leader role, women compared to men likely experience greater threat relative to challenge because of the role incongruity that follows from the stereotype of women’s lesser leadership attributes. This pattern is hypothesized to evoke a physiological stress response that includes an activation of the hypothalamic-pituitary-adrenocortical (HPA) axis (see Method for details).﻿

Research supports the idea that culturally masculine, gender-incongruent settings induce more threat than challenge for women and elicit physiological stress responses. For example, women experienced more threat relative to challenge when working in male- rather than female-dominated teams in science, technology, engineering, and mathematic (STEM) fields (Dasgupta et al., 2015). Women also showed greater activation of the physiological stress response system when watching a video of a science conference that was male-dominated, compared to one that was gender-balanced (Murphy et al., 2007).

## Feminine Role Requirements for Leadership May Benefit Women in Job Interviews

One important question is whether lack-of-fit perceptions for leader roles and the adversities that follow are inevitable for women in job interviews. According to role incongruity theories, the portrayal of leader roles as less masculine or more feminine should reduce women’s lack-of-fit perceptions (Eagly & Karau, 2002; Heilman, 1983, 2012). Empirical evidence that focused on the observer perspective has indeed shown that women suffer disadvantage primarily in the context of masculine role requirements and male-dominated roles. In fact, people evaluated women less favourably in jobs and fields that are male-dominated compared to female-dominated or integrated (e.g., Heilman et al., 2019; Koch et al., 2015). Relevant to the present research, a reframing of masculine roles can benefit women’s self-reported interest and belonging. For example, experimental research has found that reducing masculine and increasing feminine job requirements in descriptions of male-dominated roles increased women’s interest in them without harming men’s interest (Born & Taris, 2010; Gaucher et al., 2011; He & Kang, 2022). Also in STEM contexts, a rebranding of traditionally masculine roles to also afford communal values enhanced women’s anticipated belonging and interest (Belanger et al., 2020; Diekman et al., 2017).

Consistent with these empirical findings and based on the predictions from role incongruity theory, we hypothesized that emphasizing communal demands of a leader role (which are consistent with the female gender role) would attenuate women’s perceived lack of fit and its subsequent consequences without negatively affecting men. Men should not be deterred from leadership when there is an increased emphasis on feminine role requirements, given both the long history of leadership as masculine (Heilman & Manzi, 2022) and the large overlap between the stereotypical attributes of their gender role and leader roles in masculine contexts (Koenig et al., 2011). That is, we did not expect that adding feminine-typed communal role requirements to a leadership role would substantially undermine men’s experienced fit to leadership in masculine contexts as it is unlikely to seriously undermine the masculine construal of leadership.

Finally, our hiring simulation study is different from stereotype threat research in two key aspects. First, our manipulation of the gender-typing of leader role requirements targeted perceived fit to the role specifically, which is distinct from the general activation of a negative stereotype such that women would be bad leaders generally. Second, in contrast to stereotype threat research that typically featured objectively assessed performance (e.g., on complex cognitive tasks on which one’s gender stereotypically performs poorly; see reviews by Schmader et al., 2008; Spencer et al., 2016), the interviewee’s task in our hiring simulation study was self-description, which is cognitively undemanding and subjective. In fact, performance evaluations in job interviews exist in the eye of the beholder, given the absence of truly objective measures of success when self-presenting. The key outcome of our research was self- and observer-evaluated performance in an interview for a leadership position, which is different from outcomes in stereotype threat research.

## Overview and Hypotheses

This hiring simulation study featured mock interviews for a leadership role to examine if lack-of-fit perceptions affect female more than male role candidates. Overall, we hypothesized that a feminine (vs. masculine) leader role emphasis would attenuate lack-of-fit perceptions and its negative consequences for women without harming men’s experiences. The first two hypotheses thus tested the effects of participant gender on lack-of-fit processes and their consequences. The third hypothesis tested the moderating effect of the leader role framing by examining whether a feminine emphasis reduces difficulties for women candidates, without harming men candidates. The final hypothesis tested the chain of events emanating from lack-of-fit perceptions.

- Hypothesis 1: Women, compared to men, experience poorer fit for the leader role (H1a), have lower performance expectations for the interview (H1b), experience greater threat relative to challenge appraisal (H1c), show greater physiological stress responses (H1d), and evaluate their own success less favourably in an interview for a leader role (H1e).
- Hypothesis 2: Women, compared to men, receive less favourable evaluations from others of their success in an interview for a leader role.
- Hypothesis 3: A feminine, compared to a masculine, role framing attenuates the tendency of women, more than men, to experience poorer fit for the leader role (H3a), have lower performance expectations for the interview (H3b), experience greater threat relative to challenge appraisal (H3c), show greater physiological stress responses (H3d), evaluate their own success less favourably (H3e), and receive less favourable evaluations from others (H3f).
- Hypothesis 4: The poorer fit of women than men, particularly with the masculine (vs. feminine) role emphasis, elicits lower performance expectations that, in turn, elicit both greater experienced threat relative to challenge appraisal and greater physiological stress responses, and these responses, in turn, foster women’s less favourable evaluations of their performance.

## Method

### Participants and Design

The experiment had a 2 (Participant Gender: woman vs. man) × 2 (Role Framing: feminine vs. masculine) between-subjects design. Participants were management students, both undergraduate and graduate, who represent a pipeline into leadership positions. An *a priori* power analysis indicated that at least 199 participants would be adequate (1-*β* = 0.80) to detect a small effect (*f* = 0.20 or *η*^2^ = 0.039). In total, 217 students participated, with subsequent exclusions because of insufficient German skills (*n* = 4), a request for data deletion after participation (*n* = 1), or technical problems that compromised data collection (*n* = 3).

The 209 remaining participants (112 women, 97 men) ranged in age from 19 to 36 years (*M* = 24.12, *SD* = 3.15). These women and men did not differ in their number of past job interviews (*p* = .798, *η*^2^ < 0.001). Yet, the women, compared with the men, reported self-concepts that were more communal (*p* = .003, *η*^2^ = 0.043) but not less agentic (*p* = .060, *η*^2^ = 0.017; see the online Supplement A for details about the sample).

### Procedure and Materials

Email messages and flyers invited management students to participate in a study investigating students’ experience with selection procedures and including noninvasive physiological stress measurements. Following best practices, all sessions took place between 4 and 8 PM to produce consistent conditions for the physiological stress measurements (see Dickerson & Kemeny, 2004). A female and a male experimenter in business attire welcomed each participant. After giving informed consent, each participant received a compensation of CHF 50 (= USD 50). During the habituation phase of 15 minutes, the participants completed demographic and personality questionnaires. Subsequently, they provided a first saliva sample and then learned they would interview for a leadership role (see Figure S1 in the online supplement for details of the study procedure).

Participants were randomly assigned to read one of the two leader role framings and had five minutes to prepare a five-minute speech to convey that he or she was the best candidate for the job. As part of the preparation, participants wrote in their own words a description of the advertised position and the qualities expected in ideal candidates. Two research assistants independently coded whether participants (a) correctly indicated that the position was a leader role and (b) noted at least one of the role requirements or a synonym thereof. With 100% agreement between the two coders, all participants (*N* = 209) passed this manipulation check by clearly describing the role as leadership and by correctly indicating at least some of the either feminine or masculine specifics of the role.

For the job interview, which was videotaped, each participant sat in the adjacent room opposite a male experimenter, who instructed her or him to present for the role. If participants did not use the entire 5 minutes, the experimenter asked predefined questions typical of job interviews (e.g., “Why should you be chosen for this position?”), but otherwise refrained from giving verbal or nonverbal feedback.

After the interview, participants provided their second saliva sample (+ 0 min post-stress), followed by relevant questionnaires about their interview success and the threat or challenge experienced during the interview. To bridge the time until the subsequent two saliva samples and control participants’ activity, they responded to further questionnaires that asked participants about the thoughts they had during the interview (open-ended) and the attractiveness of the company (rating scales); these questionnaires were not the main focus of this research. The subsequent questions pertained to health and yielded control variables for the cortisol analyses (i.e., hormonal contraceptives, body mass index). While answering, participants were interrupted twice to provide the third saliva sample (+ 20 min post-stress) and the fourth sample (+ 40 min post-stress). Finally, the experimenter thanked and debriefed the participant.

#### Manipulation of the Framing of the Leader Role

##### Components of the Role Framing Manipulation

The construal of a leader role as more masculine or feminine is conveyed by various cues at multiple levels within an organization (see review by Schmader et al., 2020). Recent theory on masculine defaults proposed that organizational interventions are often not “fully successful because they leave in place a hidden but powerful foundation of masculine ideas and values, policies, interaction styles, norms, artifacts, practices, and individual beliefs that prevent the full participation of women” (Cheryan & Markus, 2020, p. 1022). Given that multiple cues fit together and influence each other, a powerful manipulation must tackle the framing of a leader role from multiple angles such as the gendered interaction style of ideal candidates and the representation of current leaders.

Our manipulation thus encompassed the following organizational cues to emphasize a masculine versus feminine role framing: (a) the job advertisement stated that the organization preferred candidates who were either, for example, “assertive when working with others and able to make your own decisions” (masculine) or “considerate when working with others and supportive of your team in various tasks” (feminine); (b) the header of the job advertisement depicted either a man’s or a woman’s arm holding a pen in an office space; and (c) an information sheet stated that the selection committee consisted of either two men or two women who currently occupied leader roles in the company (see Supplement B in the online supplement for transcript).

##### Check on Role Framing Manipulation

An online vignette study examined the agentic and communal requirements of the leader role with a masculine versus feminine framing. Given that organizational leadership is almost always culturally masculine and has typically been associated with agency (Koenig et al., 2011), we expected agentic requirements to prevail regardless of the role framing, whereas the communal requirements would be stronger with the feminine than the masculine framing. Supplement C in the online supplement displays the study materials and detailed methods.

In short, 305 participants (189 women, 111 men, 5 diverse) read about the leader role with either a masculine or feminine framing. Participants were enrolled in the same university as those in the hiring simulation study but had not taken part in it. On 10 items with 7-point scales that ranged from (1) *not at all* to (7) *very much*, participants rated the extent to which good role performance required agentic and communal qualities (α_Agency_ = .76, α_Communion_ = .93). As expected, a one-way ANOVA revealed that the feminine framing conveyed more communal demands than the masculine framing (*M* = 5.92, *SD* = 1.10 vs. *M* = 3.94, *SD* = 1.39), *F*(1, 303) = 129.51, *p* < .001,* η*_*p*_^*2*^ = .39. Agency was high for both role framings, yet the masculine framing conveyed more agency than the feminine framing (*M* = 6.17, *SD* = 0.72 vs. *M* = 5.49, *SD* = 0.85), *F*(1, 303) = 50.35, *p* < .001,* η*_*p*_^*2*^ = .16, but with a smaller effect size than the communion difference. 

Further tests examined whether the mean levels for required agency and required communion in the two roles were significantly above the scale midpoint. One sample *t*-tests showed that agentic requirements were significantly above the midpoint in both conditions, whereas communal requirements were above the midpoint in the feminine but not the masculine condition (see Supplement C in the online supplement for details). In sum, agentic leadership requirements were salient with both role framings, whereas the feminine emphasis added communal demands to the role.

#### Measures

##### Perceived Fit for the Leader Role

Immediately before the interview, participants assessed their perceived fit for the role on four 7-point rating scales (e.g., “I think that I am very well qualified for the advertised position;” adapted from Nater & Sczesny, 2016) that ranged from (1) *strongly disagree* to (7) *strongly agree*. The resulting scale had high internal consistency (α = .93).

##### Expected Performance

Also before the interview, participants indicated their agreement with the item “I expect to perform well in this job interview” on a 7-point scale ranging from (1) *strongly disagree* to (7) *strongly agree* (Heilman, 1983).

##### Appraisal of Threat and Challenge

After the interview, participants responded on scales ranging from 1 (*strongly disagree*) to 7 (*strongly agree*) to eight items that assessed appraisals of threat and challenge, using the Primary Appraisal Secondary Appraisal measure (PASA; Gaab, 2009). Four items assessed threat appraisal (e.g., “I felt threatened by the situation”; “I was worried because the situation was threatening to me”; α = .76), and four items assessed challenge appraisal (e.g., “The situation was relevant for me,” “The situation challenged me”; α = .65). The dependent variable was the ratio of threat to challenge, whereby a ratio greater than 1 indicated greater threat than challenge and a ratio less than 1 indicated greater challenge than threat (following Dasgupta et al., 2015).

##### Physiological Stress Response

Experiences of threat—but not of challenge—evoke cardiovascular responses that include an activation of the hypothalamic-pituitary-adrenocortical (HPA) axis, which then induces the release of the catabolic adrenal hormone cortisol (Dickerson & Kemeny, 2004). Salivary cortisol provides a valid measure of physiological stress responses in laboratory situations that elicit motivation to perform well in evaluative situations.

To allow examination of the stress responses over the course of the job interview, participants provided four salivary cortisol samples. The first sample was taken after participants’ arrival in the lab and before they learned about the leadership context (t1, baseline). Given that physiological stress responses commonly occur between 0 and 20 min after the onset of the stressor (Dickerson & Kemeny, 2004), the two subsequent samples assessed salivary cortisol levels directly after the job interview (t2) and 20 min after the interview (t3). Together with the baseline, these two samples that assessed participants’ physiological stress response were the focus of our hypotheses.

In line with the typical return to prestressor levels 40–60 min after stressor onset, the fourth sample (t4) followed 40 min after the interview and served to explore participants’ cortisol recovery (with no specific hypothesis offered). Collection of the samples used the passive drool method with IBL SaliCap tubes were stored frozen at -20 °C. Analyses of cortisol levels used time-resolved fluorimmunoassay (intraassay coefficients of variability < 5%, interassay coefficients < 8%).

##### Self-Evaluated Interview Success

First*,* on 7-point scales, participants rated themselves on 6 evaluative adjective pairs (e.g., ineffective–effective, incompetent–competent; adapted from Heilman et al., 1998). Second, on scales ranging from worst (0) to best (100) performance, participants rated themselves relative to other candidates on 5 items (e.g., “How convincingly did you present yourself as the ideal person for the position?”). Third, on a 7-point scale, participants rated their likelihood of making it to the shortlist for the position. The resulting scale of 12 standardized items had high internal consistency (α = .96).

##### Observer-Evaluated Interview Success

Four external raters (two men, two women) evaluated participants’ interview success on the same 12 items on which participants evaluated their interview success, reworded to fit the observer perspective (e.g., “How convincingly did the candidate present themselves as the ideal person for the position?”; see Supplement B in the online supplement for all items). Raters were unaware of the hypotheses and trained with practice videos. Each of the raters was randomly assigned to evaluate half of the interviewees for each leader role and participant gender. Interrater reliability was good, ICC = .82; 95% CI [.76, .86], based on a mean-rating (*k* = 4), consistency-agreement, 2-way random effects model.

### Data Analytic Strategy

#### Testing for Group Differences

A series of 2 (Participant Gender) × 2 (Role Framing) ANOVAs tested Hypotheses 1 and 2 for the predicted gender difference as well as Hypothesis 3 for the predicted attenuation of these differences with the feminine, compared to the masculine, framing.

#### Testing the Mediation Model

Testing Hypothesis 4, structural equation modeling using maximum-likelihood estimation with the *lavaan* R package (Rosseel, 2012) included participant gender as an observed variable and expected performance in the job interview and the ratio of threat versus challenge appraisal as a single indicator latent variable. The residual covariance of the two outcome latent variables was included. The tests of the specific indirect effects used 95% bias-corrected bootstrap confidence intervals based on 1,000 bootstrap samples, with unstandardized indirect effects reported. Fit was assessed by the comparative fit index (CFI), the Tucker-Lewis index (TLI), and the root-mean-square error of approximation (RMSEA; see Hu & Bentler, 1999). As shown in Supplement E in the online supplement, CFA showed a good fit to the data for all measurement models.

#### Testing Physiological Stress Responses

**Fig. 1:**
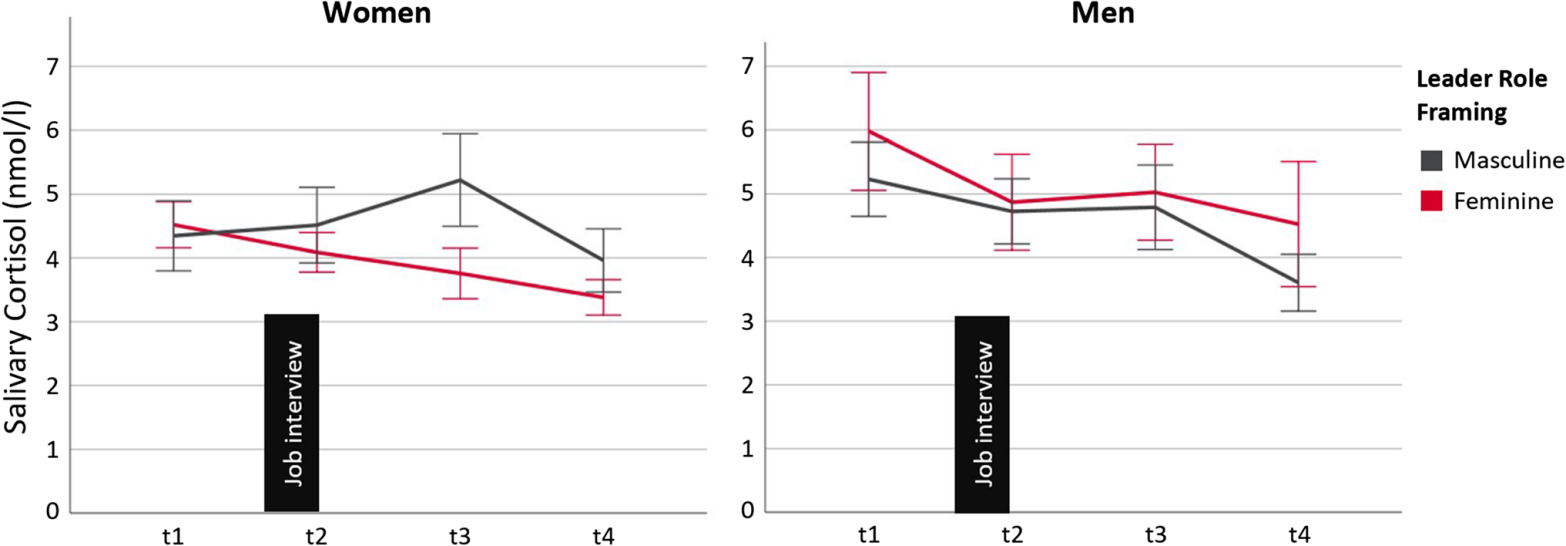
Salivary Cortisol Among Women and Men as a Function of the Framing of the Leader Role and Time of Measurement. *Note*. Salivary cortisol (nmol/l) for women (on the left) and men (on the right). The gray line indicates the cortisol change for participants presenting for the leader role with a masculine framing, and the dark red line for those presenting for the leader role with a feminine framing. Physiological stress response (Phase 1) was modeled by the t1, t2, and t3 measures, and recovery (Phase 2) by the t3 and t4 measures. The figure shows untransformed change values, whereas the analysis in the article used log-transformed data. Error bars indicate standard errors

Testing Hypotheses 1d and 3d, multilevel modeling accounted for the nested nature of the data (i.e., change in cortisol over time [Level 1] as nested within participants [Level 2]) and modeled both within-person change and between-person differences in cortisol responses (e.g., Hruschka et al., 2005). Piecewise growth modeling allowed for examining cortisol trajectories that are discontinuous (Chou et al., 2004). This study focused on participants’ stress responses evoked by the interview for the leader role, which in the piecewise growth model was modeled by the trajectory from the t1 to the t2 and t3 cortisol measures (i.e., Phase 1). Because we did not focus on gender differences in stress recovery after the job interview (modeled by the trajectory from the t3 to the t4 measure, i.e., Phase 2), these results are reported in Supplement D in the online supplement (but also displayed in Fig. 1).

A random-intercept random-slope model specified an overall intercept, two slopes (i.e., Phase 1 for physiological stress response and Phase 2 for recovery), fixed main effects of gender and framing, and the interaction of these two factors. The three-way interactions between gender, framing, and the phase variable tested the effect of the interaction between gender and framing on the slope of cortisol over the measurements. Supplement D in the online supplement displays the model equations. Implementing the R package *lme4* (Bates et al., 2015), the models were estimated by the restricted maximum-likelihood method (REML), and *t*-tests evaluated statistical significance only for fixed effects, using Satterthwaite’s method.

### Transparency and Openness

All data and analysis code are available on OSF (https://osf.io/pefc9), and the verbatim research materials in Supplement B in the online supplement. The hypotheses were not formally preregistered but were preconceived in the grant proposal that supported this research (see author note). The Ethics Commission of the University of Bern approved the study prior to data collection.

## Results

### Effects of Participant Gender and Role Framing

ANOVAs tested for gender differences and their attenuation by a feminine, compared to a masculine, framing. Table 1 shows the means and standard deviations, Table 2 the ANOVA results, and Table 3 the intercorrelations between the measures (see Table S1 in the online supplement for correlations among all study variables separated by gender).

**Table 1:** Means and Standard Deviations for the Dependent Variables, by Participant Gender (Woman vs. Man) and Role Framing (Feminine vs. Masculine)

			Perceived fit for leader role		Expected performance		Appraisal of threat and challenge		Self-evaluated interview success		Observer-evaluated interview success	
Experimental design		*N*^a^	*M*	*SD*	*M*	*SD*	*M*	*SD*	*M*	*SD*	*M*	*SD*
Women	Feminine framing	64	4.20	1.42	4.53	1.25	0.67	0.24	-0.20	0.77	-0.16	0.83
	Masculine framing	48	3.48	1.48	4.04	1.54	0.70	0.25	-0.33	0.78	-0.15	0.96
Men	Feminine framing	42	4.37	1.11	5.17	1.10	0.59	0.25	0.31	0.78	0.25	0.68
	Masculine framing	55	4.45	1.46	4.85	1.30	0.61	0.23	0.28	0.86	0.30	0.88

*Note*. The scales for perceived fit for the leader role and expected performance ranged from (1) *strongly disagree* to (7) *strongly agree*. On appraisal of threat and challenge, a ratio greater than 1 indicates participants felt more threatened than challenged, and a ratio less than 1 indicates more challenge than threat. The measures of participants’ self-evaluated and observer-evaluated interview success were standardized with higher values representing more favorable evaluations

^a^The sample for the masculine framing was reduced for self-evaluated interview success to 54 men and for observer-evaluated interview success to 47 women, due to technical problems

**Table 2:** 2 × 2 ANOVAs for the Dependent Variables as a Function of Participant Gender (Woman vs. Man) and Role Framing (Feminine vs. Masculine)

Source	*df*	*F*	*p*	*η*_*p*_^*2*^
Perceived fit for leader role				
Gender	1	14.73	< .001	.067
Framing	1	6.60	.011	.031
Framing × Gender	1	1.26	.264	.006
Error	205			
Expected performance				
Gender	1	15.64	< .001	.071
Framing	1	4.79	.030	.023
Framing × Gender	1	0.24	.629	.001
Error	205			
Appraisal of threat versus challenge				
Gender	1	6.32	.013	.030
Framing	1	0.64	.426	.003
Framing × Gender	1	0.06	.810	< .001
Error	205			
Self-evaluated interview success				
Gender	1	25.34	< .001	.110
Framing	1	0.54	.462	.003
Framing × Gender	1	0.22	.641	.001
Error	205			
Observer-evaluated interview success				
Gender	1	12.86	< .001	.059
Framing	1	0.08	.784	< .001
Framing × Gender	1	0.02	.882	< .001
Error	204

**Table 3:** Pearson Correlations

Variables	1	2	3	4	5	6	7
1. Participant gender	–						
2. Framing of leader role	-.14*	–					
3. Perceived qualifications	-.23***	-.14*	–				
4. Expected performance	-.25***	-.11	.64***	–			
5. Appraisal of threat vs. challenge	.17*	.03	-.26***	-.33***	–		
6. Physiological stress response (AUCi)	.14	.16*	.02	.06	.06	–	
7. Self-evaluated interview success	-.33***	-.01	.51***	.57***	-.48***	-.10	–
8. Observer-evaluated interview success	-.25***	.05	.17*	.18**	-.26***	-.08	.39***

*Note*. Participant gender was coded man = 0, woman = 1; Framing of leader role was coded feminine = 0, masculine = 1. For the correlational analyses, physiological stress responses (i.e., Phase 1; assessed by t1, t2, and t3 cortisol measures) was mapped onto one variable by calculating the area-under-the-curve with regard to the increase (AUC_i_; Pruessner et al., 2003)

^*^ for *p* < *.*05; ** for *p* < .01; *** for *p* < .001

As shown in Table 2, and consistent with our prediction, women, compared to men, experienced less fit for the leader role (Hypothesis 1a), expected they would perform more poorly in the interview (Hypothesis 1b), experienced greater threat relative to challenge appraisal (Hypothesis 1c), and evaluated their own interview success less favourably (Hypothesis 1e). Results on the physiological stress response (Hypothesis 1d) did not support the prediction that women, compared to men, would experience greater physiological stress overall (see next section). Additionally consistent with our predictions, a significant main effect of participant gender indicated that women, compared to men, received less favorable interview success evaluations according to others’ judgments (Hypothesis 2).

Although not hypothesized, the ANOVA further revealed significant role framing main effects, indicating that the feminine role framing produced significantly more favourable fit perceptions and expected performance for both women and men. However, the role framing did not affect the appraisal of threat relative to challenge or evaluations of interview success. Moreover, exploratory analyses revealed that when threat and challenge were disaggregated as separate dependent variables, the main effect of gender was significant for threat but not for challenge, indicating that women felt more threatened but not more challenged than men in the job interview (see Hypothesis 1c; see Tables S2 and S3 in the online supplement).

Contrary to predictions that the gender differences would be attenuated in the presence of a feminine, compared to a masculine, role framing (Hypothesis 3), the Participant Gender × Role Framing interactions were nonsignificant for self-reported fit perceptions, performance expectations, threat relative to challenge appraisals, as well as for self-evaluations and others’ evaluations of performance. These results thus failed to support predictions that the feminine framing would attenuate women’s difficulties relative to those of men as reported by participants themselves. Importantly, however, the physiological stress responses (Hypothesis 3d) yielded a significant three-way Role Framing × Gender × Phase 1 interaction that provided support for our prediction (see next section).

### Physiological Stress Responses

Multilevel analyses modeled estimates for the effects of gender and role framing on cortisol trajectories. Initial analyses showed that across participants and conditions, average cortisol levels were highest at the onset of the experiment (t1 baseline; *M* = 4.96, *SD* = 4.25), indicating high anticipatory stress levels before the job interview. However, only for the women in the masculine framing condition were the average cortisol levels highest at the peak stress measure (t3, taken 20 min after the interview; *M* = 5.22, *SD* = 5.01). A random intercept-only model revealed an intraclass correlation coefficient (ICC) of 80.46%, indicating that a large part of the variance in cortisol levels is attributable to differences between participants (i.e., level 2).

As outlined in the Method, hypothesis tests focused on physiological stress responses elicited by the job interview situation (i.e., Phase 1 in the piecewise growth model, assessed by the t1, t2, t3 measures). Supplement D in the online supplement displays the results for stress recovery (i.e., Phase 2), indicating that the framing did not differentially affect women’s and men’s recovery. Table 4 shows mean values and standard deviations for the cortisol levels at the four measurement points. Table 5 shows the piecewise growth modeling estimates for the effects of gender and leader role framing. Figure 1 displays the trajectories of salivary cortisol for women and men in the two role framing conditions.

**Table 4:** Means and Standard Deviations for Cortisol Measures, by Participant Gender and Role Framing

			Physiological stress response							
			t1		t2		t3		t4	
Experimental design		*N*^*a*^	*M*	*SD*	*M*	*SD*	*M*	*SD*	*M*	*SD*
Women	Feminine Frame	64	4.52	2.90	4.09	2.49	3.76	3.18	3.38	2.22
	Masculine Frame	48	4.35	3.81	4.52	4.11	5.22	5.01	3.96	3.44
Men	Feminine Frame	42	5.98	5.99	4.87	4.88	5.03	4.89	4.52	6.37
	Masculine Frame	55	5.23	4.32	4.73	3.79	4.79	4.93	3.60	3.31

*Note*. Physiological stress responses are expressed as cortisol units nmol/l

^a^The sample for the masculine framing was reduced for self-evaluated interview success to 54 men and for observer-evaluated interview success to 47 women, due to technical problems

**Table 5:** Model Estimates for the Prediction of Cortisol Levels as a Function of Participant Gender and Role Framing

Variables	Coefficients (*SE*)	*df*	*t*	*p*
**Fixed effect﻿s**				
Intercept	1.24 (0.13)	205.11	9.39	< .001
Phase 1 (Stress)	-0.03 (0.05)	205.90	-0.70	.484
Phase 2 (Recovery)	-0.20 (0.06)	384.75	-3.58	< .001
Gender	-0.20 (0.17)	205.11	-1.16	.247
Framing	-0.10 (0.18)	205.11	-0.58	.560
Gender × Framing	0.27 (0.24)	205.11	1.11	.268
Gender × Phase 1	-0.11 (0.06)	205.90	-1.86	.065
Gender × Phase 2	0.16 (0.07)	384.75	2.20	.028
Framing × Phase 1	-0.06 (0.06)	205.90	-0.96	.339
Framing × Phase 2	-0.02 (0.08)	384.75	-0.22	.825
Gender × Framing × Phase 1	0.20 (0.08)	205.90	2.43	.016
Gender × Framing × Phase 2	-0.12 (0.10)	384.75	-1.14	.257
**Random effects**	Estimate (*SD*)			
Random intercept	0.68 (0.82)			
Random slope Phase 1	0.05 (0.23)			
Random slope Phase 2	0.003 (0.06)			
Residual variance	0.07 (0.27)			

*Note*. Participant gender was coded man = 0, woman = 1; Framing of leader role was coded feminine = 0, masculine = 1. Based on 209 participants with 836 longitudinal records. Phase 1 = Physiological stress responses modeled by the t1, t2, t3 measure; Phase 2 = Recovery modeled by the t3 and t4 cortisol measures; *SE* = standard errors; Estimate (*SD*) = Estimated variance of the random effect with the square root of that variance (i.e., sigma) in brackets. Unstandardized regression coefficients are reported

As shown in Table 5, supporting Hypothesis 3d, results indicated a significant three-way Role Framing × Gender × Phase 1 interaction. Decomposition of this interaction within levels of gender found a significant Role Framing × Phase 1 simple interaction for women, *B* = 0.14, *SE* = 0.06, *df* = 205.90,* t* = 2.53, *p* = .012, but not for men, *B* = -0.06, *SE* = 0.06, *df* = 205.90,* t* = 0.96, *p* = .339, indicating that only the women showed stronger physiological stress responses in the masculine than feminine condition, whereas no such difference emerged for the men (see Fig. 1).

Decomposing this Role Framing × Phase 1 simple interaction for women within levels of framing found a nonsignificant positive slope over time for the masculine condition, *B* = 0.002, *SE* = 0.04, *df* = 205.90,* t* = 0.05, *p* = .961, and a significant negative slope over time for the feminine condition, *B* = -0.14, *SE* = 0.04, *df* = 205.90,* t* = -3.82, *p* < .001, documenting reduced stress responses among women only in the role with a feminine, but not a masculine, framing. 

As robustness checks, two repetitions of the model that controlled cortisol levels for the potentially relevant confounds of BMI or women’s hormonal contraceptives (i.e., 65.2% used versus 34.8% did not use). By adding one variable each to the between-subjects level of the MLM model, the covariates were controlled as level-2 fixed effects in separate models. These models produced similar results as the uncontrolled model, and the Role Framing × Gender × Phase 1 interaction remained significant.^1^

Finally, testing Hypothesis 1d that women compared to men showed greater physiological stress responses (Phase 1) regardless of the role framing, contrast analyses found no significant overall gender difference, *B* = 0.02 *SE* = 0.04, *df* = 207.00,* t* = 0.42, *p* = .676.

### Underlying Mechanism Explaining Gender Differences in Interview Self-Evaluation

Finally, structural equation modeling examined whether gender differences in evaluated interview success—particularly for the leader role with a masculine frame—were fuelled by women’s relatively lower perceived fit (see Hypothesis 4). These perceptions then should relate to women’s lower expected performance for the interview, which in turn should relate to both greater threat relative to challenge appraisal and greater physiological stress response, and subsequently to women’s less favourable self-evaluated interview success.

**Fig. 2:**
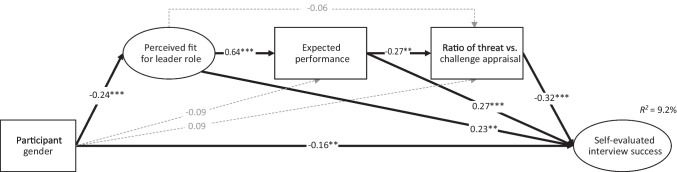
Structural Equation Model of the Effect of Participant Gender on Self-Evaluated Interview Success Through Perceived Fit for Leader Role, Expected Performance in Job Interview, and Threat Versus Challenge Appraisal. *Note*. Standardized results are depicted. Significant paths are indicated by a solid line, and nonsignificant paths by a dashed line. Participant gender was coded man = 0, woman = 1. Ellipses represent latent variables, and rectangles represent observed variables. * for *p* < .05; ** for *p* < .01; *** for *p* < .001

Although our hypothesis originally included physiological stress as a mediator and role framing as a moderator, we present a more parsimonious model (see Fig. 2). However, Supplement E in the online supplement displays the originally proposed moderated mediation model, which showed that the physiological stress responses did not mediate any effect, (b) the inclusion did not substantially change the model fit, and (c) the leader role framing did not moderate any structural paths. The decision to change the model was thus informed by the results so far showing that (a) physiological stress responses were unrelated to any of the self-report measures^2^ (see Table 3), and (b) leader role framing did not moderate any of the self-reported or the other-evaluated outcomes (see Hypotheses 3a-3c and 3e-3f).

Results showed a good model fit, *χ*^*2*^(145) = 317.21, *p* < .001; CFI = .950; TLI = .942; RMSEA = .075, 90% CI [0.064, 0.087], accounting for 49.1% of the variance in self-evaluated interview success. Supporting Hypothesis 4, an indirect effect of gender on self-evaluation (-.03, 95% CI [-0.08, -0.01]) showed that women’s compared to men’s lower perceived fit, *b* = -0.24, *p* < .001, related to lower expected performance for the interview, *b* = 0.64, *p* < .001, which in turn related to greater threat versus challenge appraisal, *b* = -0.27, *p* = .002, and subsequently to women’s poorer self-evaluated success, *b* = -0.32, *p* < .001 (see Fig. 2).

Consistent with the process predicted by incongruity theory, two additional indirect effects emerged. First, as stated in the lack of fit model (see Heilman, 1983, p. 279), women’s compared to men’s lower fit perceptions and subsequent lower expected performance for the interview related to women’s less favourable self-evaluated success (*b* = -.10, 95% CI [-0.22, -0.04]). In addition, women’s compared to men’s lower fit perceptions related directly to their less favourable self-evaluation (*b* = -.08, 95% CI [-0.19, -0.03]). Finally, the direct effect of gender on self-evaluation remained significant, *b* = -0.16, *p* = .003.

## Discussion

This hiring simulation study examined how lack-of-fit perceptions for a leader role caused women to have different experiences than men in a job interview for such a role. The results showed that a framing intervention that added feminine qualities to a leader role’s typical emphasis on masculine qualities reduced the physiological stress responses of the women, but not the men, job candidates. However, regardless of the role framing, women self-reported lesser fit than men and expected to perform less successfully, and these effects related to greater threat versus challenge appraisal and subsequently less favourable performance evaluations by others and the candidates themselves. These less favorable self-evaluations are posited by incongruity theories (e.g., Heilman, 1983) but have, to our knowledge, not been previously tested. Also, additional exploratory mediational findings supported incongruity theories by showing that women’s lower level of self-ascribed agency, assessed before participants learned about the goal of the study, was a precursor of their lower fit perceptions (see Supplement E in the online supplement).

Lack-of-fit perceptions and the subsequent difficulties women can face in interviews for leader roles are consequential because interviews function as a gateway to occupational positions. Given that a job interview for leadership, with its typical demand for assertive self-promotion, can emphasise the masculine demands of a leader role (Cheryan & Markus, 2020), interventions aiming to lessen the disadvantage of women should consider limiting masculine cues and highlighting feminine cues in descriptions of leader roles and job interviews. Reducing cues for assertion, ambition, and risk-taking while increasing cues for of consideration, social responsibility, and cooperation could alleviate women’s otherwise greater physiological stress responses. However, given our findings, such interventions may not be sufficient to stave off unfavourable evaluations of women’s interview performance.

By using live social interactions and multiple outcome measures, this hiring simulation study uncovered novel effects of role incongruity. Specifically, the objective stress biomarker salivary cortisol revealed that the leader role with a feminine (vs. masculine) framing reduced women’s physiological stress responses during self-presentation, without increasing men’s. This effect, which differs from the self-report results, coheres with research typically showing noncorrespondence between physiological and self-reported measures of stress. In fact, Campbell and Ehlert (2012) documented noncorrespondence in 75% of the examined studies, perhaps due to differences in the measures’ reliance on conscious attention and the measures' dependence on the motivation to give accurate self-reports.

Supporting these possibilities, research on explicit versus implicit attitudes found that implicit measures of prejudice better predicted spontaneous and automatic behaviour (e.g., non verbal friendliness), whereas self-reported explicit measures better predicted conscious, deliberative, and controlled behaviour (e.g., verbal behaviour; Dovidio et al., 2002). Relatedly, in our study, the role framing with an emphasis on feminine requirements mitigated women’s spontaneous physiological stress responses, whereas no such beneficial effect occurred on responses requiring conscious attention and contemplation. Thus, a reframing of leader role requirements may benefit women (without harming men) on a more unconscious physiological level, but not on responses requiring conscious reflection and deliberative responding, with the latter potentially being open to influences of ingrained beliefs about women and their incongruity with leadership.

Consistent with our physiological stress findings, research has found that female managers experienced higher physiological stress in general than male managers (Lundberg & Frankenhaeuser, 1999). Against this background, it is important that the repeated activation of the HPA system and subsequent cortisol release related to various negative health outcomes such as impaired immune functions and depressive symptomatology (Derks & Scheepers, 2018; Dickerson & Kemeny, 2004). Therefore, if the benefits of feminine role emphasis for stress responses found in this simulation study generalize to organizational settings, increasing the salience of feminine role requirements of leadership could lessen some of female managers’ health challenges.

### Limitations and Future Research Directions

Limitations of the present research and its findings raise questions for further inquiry. First, to fully examine stress responses and performance evaluations in job interviews, it is important to replicate this work in real-life interviews to determine whether the effects in our controlled experimental study prevail in natural settings in which (nonverbal) feedback from the interviewer is available on a continuous basis. Future research should further focus on whether women’s responses depend on their actual interest in securing a leadership position during a job interview, given that past work has shown that personal motivation matters. Relevant studies by Hoyt and Blascovich (2007, 2010) examined women with either particularly high or low leadership self-efficacy and found that women with high levels, for whom the leadership task was self-relevant, experienced threat during the task, yet performed better and reacted against the leader-gender stereotype when they had been primed (vs. not primed) with it. Applying this knowledge to the job interview situation, future research should test whether women highly invested in becoming a leader would show greater threat responses and possibly also perform better, especially when interviewing for a masculine-framed leader role.

#### Multiple Origins of Women’s Stress Experience and Performance Outcomes

In a job interview situation with live social interaction, women’s experiences of stress may have origins other than the difficulties arising from role incongruity and the masculine construal of leadership (Eagly & Karau, 2002; Heilman, 1983, 2012). Specifically, women more than men may have been concerned about gender bias in leadership hiring (e.g., Hardy et al., 2022) or feared backlash from others for engaging in self-promotion (Moss-Racusin & Rudman, 2010). In fact, any research with live social interactions and job interviews might provide a conservative test of a manipulation of leader role requirements, given that the interview itself required self-promotion. Doing well in an interview entails promoting oneself assertively, behavior that is inconsistent with feminine norms of modesty and concern for others (Rudman & Phelan, 2008). Women may therefore feel poorly equipped for success in an interview regardless of the framing of the leader role for which they are applying.

Moreover, another process that could have contributed to women’s greater stress and impaired performance evaluations is stereotype threat, which occurs when individuals show decreased task performance in a domain in which their group is negatively stereotyped (Schmader et al., 2008). Yet, women may not be stereotyped as bad leaders per se, as illustrated by the public discourse that often claims that women would be better leaders than men (e.g., Chamorro-Premuzic, 2021). By manipulating whether the evaluator holds or refutes beliefs that women are bad leaders, future research could test how participants' fear of the negative stereotype about their group affects them. Furthermore, future work should test whether the underlying mechanism found to impair subjective evaluations of performance carried out by our participants and the external observers would also impair women’s performance on tasks that allow for fully objective evaluations.

In sum, future research is necessary to distinguish between these likely intertwined processes (i.e., role incongruity, concern about gender bias, fear of backlash, stereotype threat)—to the extent this is possible. Such research would help to determine if there are conditions under which feminine framing can have the predicted beneficial effects on self- and other-reported performance evaluation due to relaxation of lack-of-fit perceptions. To accomplish this, it would be helpful to investigate (objective) performance outcomes in settings other than interviews.

#### The Omnipresence of Agency in Leadership

Contrary to our predictions, results indicated that the feminine role intervention did not diminish women’s self-reported lack of fit, greater threat than challenge appraisal, or lower success expectation for the interview. As shown by the role framing manipulation check (see Method), the emphasis on feminine role requirements increased the role’s communal demands, yet agentic demands remained prevalent. In fact, our manipulation made the leader role more androgynous (i.e., fairly equally masculine and feminine) but did not relax the masculine construal of leadership. This finding aligns with the idea that leadership is ordinaily masculine and that agentic demands are inherent in virtually all organizational leader roles (e.g., Koenig et al., 2011).

The specific contours of role incongruity for female leaders also are worthy of consideration. For example, future research should examine the effects of different types of agency (e.g., ambitious agency, dominant agency; see Ma et al., 2022), which may differ in their propensity to produce lack-of-fit perceptions. Relatedly, future work may test whether a lowering of agentic demands rather than an enhancement of communal demands would better alleviate difficulties that women face in job interviews.

### Practice Implications

Understanding women’s experiences during job interviews for leader roles is important, given that self-evaluations of performance can influence subsequent motivation and work aspirations (Kim et al., 2010), thereby subtly influencing the course of one’s career trajectory. Such knowledge is furthermore crucial to enabling women’s success in this pivotal moment on the way to a leadership career because early derailment in interviews is no doubt one cause of women’s persistent underrepresentation in masculine-typed leadership.

Women’s lack-of-fit perceptions derive from both beliefs about the leadership role and the female gender role. Either of these factors can provide leverage for change. Promising interventions may alter the requirements of leader roles to achieve a better fit with culturally feminine attributes. However, as our research makes clear, successfully mitigating the prevailing masculine characterization of leadership is no easy task, given its deep roots in society (e.g., Eagly & Koenig, 2021). Given this phenomenon, increasing women’s sense of agency might seem to be a straightforward solution—as illustrated by calls for women to “lean in” and be assertive to tackle their underrepresentation (e.g., Sandberg, 2013). Yet, although our results suggest that an agentic self-concept can potentially deter the sequence of events demonstrated here, this possibility ignores the individual and structural barriers that complicate women’s attainment of leadership positions (also see review by Eagly, 2018).

Nevertheless, many women do attain leadership positions and achieve success as leaders. This fact refutes the claim that women are simply less fit for leadership. However, it is notable that although women now occupy an impressive share of leader roles, including, for example, 28% of computer and information systems managers, most women leaders are in domains considered to be feminine in gender type (e.g., 72% of social and community service managers; U.S. Bureau of Labor Statistics, 2023)—domains that are unlikely to elicit lack-of-fit perceptions. These data indicate that women are capable of being strong and effective leaders but also imply that their acceptance into leadership roles may be best in industries and occupations for which they appear to be a ‘good fit.’

Lastly, our results revealed an unanticipated benefit for women and men of a leader role not explicitly defined in masculine terms. In fact, the leader role with a feminine framing improved both women’s and men’s assessment of their fit and performance expectations for the interview. This finding aligns with research showing that, even among children, both girls and boys showed greater interest when a leader role was feminine-framed in terms of helping others rather than masculine-framed without this expectation (Vial & Cimpian, 2024). Thus, increasing the feminine framing of leader roles may benefit confidence to do the task better among both genders.

## Conclusion

This simulation of a job interview for a leadership position showed that women’s physiological stress responses were alleviated by a framing intervention that added feminine qualities to a leadership role’s typical emphasis on masculine qualities. Yet, in terms of self-reported fit perceptions, expected performance, threat versus challenge appraisal, and self-evaluations of interview success, the role framing with a feminine emphasis did not erase women’s less favourable outcomes in the interview. Our findings thus provide compelling evidence of the difficulties of dislodging masculine defaults in leadership, as in other aspects of society.

## Supplementary Information

Below is the link to the electronic supplementary material.

Supplementary file1 (DOCX 2035 KB)

## Data Availability

All data, analysis code, and research materials are available at OSF (https://osf.io/pefc9). This research was supported by two grants from the Swiss National Science Foundation awarded to Christa Nater (grant P0BEP1_162210) and to Sabine Sczesny and Nadine Messerli-Bürgy (grant PDFMP1_137144).

## Footnotes

1 The Framing × Phase 1 interaction for the women remained significant when controlling for BMI and became a near-significant trend when controlling for contraceptive usage.

2 Given the absence of a linear relationship between cortisol responses and performance, we explored whether a curvilinear relationship exists with moderate levels of cortisol response, but not extreme levels, relating to peak performance (drawing upon the inverted-U-shape relation between arousal and performance in basic cognitive task, e.g., Beerendonk et al., 2024). Results found that the nonlinear model did not explain a significantly larger amount of variance than the linear model, for the relationship between cortisol response (i.e., AUC_i_, see Table 3 note for details) and both performance self-evaluation, *F*(206) = 143.85, *p* = .054, and performance other-evaluation, *F*(205) = 154.85, *p* = .995.

## References

[CR1] Bates, D., Mächler, M., Bolker, B., & Walker, S. (2015). Fitting linear mixed-effects models using lme4. *Journal of Statistical Software,**67*, 1–48. 10.18637/jss.v067.i01

[CR2] Beerendonk, L., Mejías, J. F., Nuiten, S. A., de Gee, J. W., Fahrenfort, J. J., & van Gaal, S. (2024). A disinhibitory circuit mechanism explains a general principle of peak performance during mid-level arousal. *Proceedings of the National Academy of Sciences,**121*(5), e2312898121. 10.1073/pnas.2312898121 10.1073/pnas.2312898121 PMC10835062 38277436

[CR3] Belanger, A. L., Joshi, M. P., Fuesting, M. A., Weisgram, E. S., Claypool, H. M., & Diekman, A. B. (2020). Putting belonging in context: Communal affordances signal belonging in STEM. *Personality and Social Psychology Bulletin,**46*, 1186–1204. 10.1177/0146167219897181 31928327 10.1177/0146167219897181 PMC7996047

[CR4] Blascovich, J., & Tomaka, J. (1996). The biopsychosocial model of arousal regulation. In M. P. Zanna (Ed.), *Advances in experimental social psychology* (Vol. 28, pp. 1–51). Academic Press. 10.1016/S0065-2601(08)60235-X

[CR5] Born, M. P., & Taris, T. W. (2010). The impact of the wording of employment advertisements on students’ inclination to apply for a job. *Journal of Social Psychology,**150*, 485–502. 10.1080/00224540903365422 21058576 10.1080/00224540903365422

[CR6] Bosak, J., & Sczesny, S. (2008). Am I the right candidate? Self-ascribed fit of women and men to a leadership position. *Sex Roles,**58*, 682–688. 10.1007/s11199-007-9380-4

[CR7] Campbell, J., & Ehlert, U. (2012). Acute psychosocial stress: Does the emotional stress response correspond with physiological responses? *Psychoneuroendocrinology,**37*, 1111–1134. 10.1016/j.psyneuen.2011.12.010 22260938 10.1016/j.psyneuen.2011.12.010

[CR8] Carli, L. L. (2018). Women, power, and the career labyrinth. In *APA handbook of the psychology of women: Perspectives on women’s private and public lives, Vol. 2* (pp. 349–365). American Psychological Association. 10.1037/0000060-019

[CR9] Chamorro-Premuzic, T. (2021, December 10). If women are better leaders, then why are they not in charge? *Forbes*. https://www.forbes.com/sites/tomaspremuzic/2021/03/07/if-women-are-better-leaders-then-why-are-they-not-in-charge/

[CR10] Cheryan, S., & Markus, H. R. (2020). Masculine defaults: Identifying and mitigating hidden cultural biases. *Psychological Review,**127*, 1022–1052. 10.1037/rev0000209 32804526 10.1037/rev0000209

[CR11] Chou, C.-P., Yang, D., Pentz, M. A., & Hser, Y.-I. (2004). Piecewise growth curve modeling approach for longitudinal prevention study. *Computational Statistics & Data Analysis,**46*, 213–225. 10.1016/S0167-9473(03)00149-X

[CR12] Dasgupta, N., Scircle, M. M., & Hunsinger, M. (2015). Female peers in small work groups enhance women’s motivation, verbal participation, and career aspirations in engineering. *Proceedings of the National Academy of Sciences of the United States of America,**112*, 4988–4993. 10.1073/pnas.1422822112 25848061 10.1073/pnas.1422822112 PMC4413283

[CR13] Derks, B., & Scheepers, D. (2018). Neural and cardiovascular pathways from stigma to suboptimal health. In B. Major, J. F. Dovidio, & B. G. Link (Eds.), *The Oxford Handbook of Stigma, Discrimination, and Health* (pp. 241–264). Oxford University Press. 10.1093/oxfordhb/9780190243470.013.9

[CR14] Dickerson, S. S., & Kemeny, M. E. (2004). Acute stressors and cortisol responses: A theoretical integration and synthesis of laboratory research. *Psychological Bulletin,**130*, 355–391. 10.1037/0033-2909.130.3.355 15122924 10.1037/0033-2909.130.3.355

[CR15] Diekman, A. B., Steinberg, M., Brown, E. R., Belanger, A. L., & Clark, E. K. (2017). A goal congruity model of role entry, engagement, and exit: Understanding communal goal processes in STEM gender gaps. *Personality and Social Psychology Review,**21*(2), 142–175. 10.1177/1088868316642141 27052431 10.1177/1088868316642141

[CR16] Dovidio, J. F., Kawakami, K., & Gaertner, S. L. (2002). Implicit and explicit prejudice and interracial interaction. *Journal of Personality and Social Psychology,**82*, 62–68. 10.1037/0022-3514.82.1.62 11811635 10.1037//0022-3514.82.1.62

[CR17] Eagly, A. H. (2018). The shaping of science by ideology: How feminism inspired, led, and constrained scientific understanding of sex and gender. *Journal of Social Issues,**74*, 871–888. 10.1111/josi.12291

[CR18] Eagly, A. H., & Karau, S. J. (2002). Role congruity theory of prejudice toward female leaders. *Psychological Review,**109*, 573–598. 10.1037/0033-295X.109.3.573 12088246 10.1037/0033-295x.109.3.573

[CR19] Eagly, A. H., & Koenig, A. M. (2021). The vicious cycle linking stereotypes and social roles. *Current Directions in Psychological Science,**30*, 343–350. 10.1177/09637214211013775

[CR20] Eagly, A. H., Makhijani, M. G., & Klonsky, B. G. (1992). Gender and the evaluation of leaders: A meta-analysis. *Psychological Bulletin,**111*, 3–22. 10.1037/h0090375 10.1037/0033-2909.117.1.125 7870858

[CR21] Eagly, A. H., Nater, C., Miller, D. I., Kaufmann, M., & Sczesny, S. (2020). Gender stereotypes have changed: A cross-temporal meta-analysis of U.S. public opinion polls from 1946 to 2018. *American Psychologist,**75*, 301–315. 10.1037/amp0000494 31318237 10.1037/amp0000494

[CR22] Gaab, J. (2009). PASA: Primary appraisal secondary appraisal. *Verhaltenstherapie,**19*, 114–115. 10.1159/000223610

[CR23] Gaucher, D., Friesen, J., & Kay, A. C. (2011). Evidence that gendered wording in job advertisements exists and sustains gender inequality. *Journal of Personality and Social Psychology,**101*, 109–128. 10.1037/a0022530 21381851 10.1037/a0022530

[CR24] Hardy, J. H., Tey, K. S., Cyrus-Lai, W., Martell, R. F., Olstad, A., & Uhlmann, E. L. (2022). Bias in context: Small biases in hiring evaluations have cig consequences. *Journal of Management,**48*, 657–692. 10.1177/0149206320982654

[CR25] He, J. C., & Kang, S. K. (2022). Identities between the lines: Re-aligning gender and professional identities in job advertisements. *Academy of Management Proceedings*. 10.5465/AMBPP.2022.10415abstract

[CR26] Heilman, M. E. (1983). Sex bias in work settings: The lack of fit model. *Research in Organizational Behavior,**5*, 269–298.

[CR27] Heilman, M. E. (2012). Gender stereotypes and workplace bias. *Research in Organizational Behavior,**32*, 113–135. 10.1016/j.riob.2012.11.003

[CR28] Heilman, M. E., & Manzi, F. (2022). Revisiting Schein’s think manager-think male study. In N. K. Steffens, F. Rink, & M. K. Ryan (Eds.), *Organisational psychology: Revisiting the classic studies* (pp. 221–240). SAGE.

[CR29] Heilman, M. E., Battle, W. S., Keller, C. E., & Lee, R. A. (1998). Type of affirmative action policy: A determinant of reactions to sex-based preferential selection? *Journal of Applied Psychology,**83*, 190–205. 10.1037/0021-9010.83.2.190 9577233 10.1037/0021-9010.83.2.190

[CR30] Heilman, M. E., Manzi, F., & Caleo, S. (2019). Updating impressions: The differential effects of new performance information on evaluations of women and men. *Organizational Behavior and Human Decision Processes,**152*, 105–121. 10.1016/j.obhdp.2019.03.010

[CR31] Heilman, M. E., Caleo, S., & Manzi, F. (2024). Women at work: Pathways from gender stereotypes to gender bias and discrimination. *Annual Review of Organizational Psychology and Organizational Behavior,**11*, 165–192. 10.1146/annurev-orgpsych-110721-034105

[CR32] Hoyt, C. L., & Blascovich, J. (2007). Leadership efficacy and women leaders’ responses to stereotype activation. *Group Processes & Intergroup Relations,**10*, 595–616. 10.1177/1368430207084718

[CR33] Hoyt, C. L., & Blascovich, J. (2010). The role of leadership self-efficacy and stereotype activation on cardiovascular, behavioral and self-report responses in the leadership domain. *The Leadership Quarterly,**21*, 89–103. 10.1016/j.leaqua.2009.10.007

[CR34] Hruschka, D. J., Kohrt, B. A., & Worthman, C. M. (2005). Estimating between- and within-individual variation in cortisol levels using multilevel models. *Psychoneuroendocrinology,**30*, 698–714. 10.1016/j.psyneuen.2005.03.002 15854786 10.1016/j.psyneuen.2005.03.002

[CR35] Hsu, N., Badura, K. L., Newman, D. A., & Speach, M. E. P. (2021). Gender, “masculinity”, and “femininity”: A meta-analytic review of gender differences in agency and communion. *Psychological Bulletin,**147*, 987–1011. 10.1037/bul0000343

[CR36] Hu, L., & Bentler, P. M. (1999). Cutoff criteria for fit indexes in covariance structure analysis: Conventional criteria versus new alternatives. *Structural Equation Modeling: A Multidisciplinary Journal,**6*, 1–55. 10.1080/10705519909540118

[CR37] Kim, Y.-H., Chiu, C., & Zou, Z. (2010). Know thyself: Misperceptions of actual performance undermine achievement motivation, future performance, and subjective well-being. *Journal of Personality and Social Psychology,**99*, 395–409. 10.1037/a0020555 20804261 10.1037/a0020555

[CR38] Koch, A. J., D’Mello, S. D., & Sackett, P. R. (2015). A meta-analysis of gender stereotypes and bias in experimental simulations of employment decision making. *Journal of Applied Psychology,**100*, 128–161. 10.1037/a0036734 24865576 10.1037/a0036734

[CR39] Koenig, A. M., Eagly, A. H., Mitchell, A. A., & Ristikari, T. (2011). Are leader stereotypes masculine? A meta-analysis of three research paradigms. *Psychological Bulletin,**137*, 616–642. 10.1037/a0023557 21639606 10.1037/a0023557

[CR40] Lundberg, U., & Frankenhaeuser, M. (1999). Stress and workload of men and women in high-ranking positions. *Journal of Occupational Health Psychology,**4*, 142–151. 10.1037/1076-8998.4.2.142 10212866 10.1037//1076-8998.4.2.142

[CR41] Lyness, K. S., & Grotto, A. R. (2018). Women and leadership in the United States: Are we closing the gender gap? *Annual Review of Organizational Psychology and Organizational Behavior,**5*, 227–265. 10.1146/annurev-orgpsych-032117-104739

[CR42] Ma, A., Rosette, A. S., & Koval, C. Z. (2022). Reconciling female agentic advantage and disadvantage with the CADDIS measure of agency. *Journal of Applied Psychology,**107*, 2115–2148. 10.1037/apl0000550 35298212 10.1037/apl0000550

[CR43] Moss-Racusin, C. A., & Rudman, L. A. (2010). Disruptions in women’s self-promotion: The backlash avoidance model. *Psychology of Women Quarterly,**34*, 186–202. 10.1111/j.1471-6402.2010.01561.x

[CR44] Murphy, M. C., Steele, C. M., & Gross, J. J. (2007). Signaling threat: How situational cues affect women in math, science, and engineering settings. *Psychological Science,**18*, 879–885. 10.1111/j.1467-9280.2007.01995.x 17894605 10.1111/j.1467-9280.2007.01995.x

[CR45] Nater, C., & Sczesny, S. (2016). Affirmative action policies in job advertisements for leadership positions: How they affect women’s and men’s inclination to apply. *European Journal of Social Psychology,**46*, 891–902. 10.1002/ejsp.2200

[CR46] Netchaeva, E., Sheppard, L. D., & Balushkina, T. (2022). A meta-analytic review of the gender difference in leadership aspirations. *Journal of Vocational Behavior,**137*, 103744. 10.1016/j.jvb.2022.103744

[CR47] Peterson, S. J., & Bartels, A. L. (2017). Using neuroscience methods to explore gender differences in leadership. In S. R. Madsen (Ed.), *Handbook of research on gender and leadership* (pp. 238–253). Edward Elgar Publishing. 10.4337/9781785363863.00024

[CR48] Pruessner, J. C., Kirschbaum, C., Meinlschmid, G., & Hellhammer, D. H. (2003). Two formulas for computation of the area under the curve represent measures of total hormone concentration versus time-dependent change. *Psychoneuroendocrinology,**28*(7), 916–931. 10.1016/S0306-4530(02)00108-7 12892658 10.1016/s0306-4530(02)00108-7

[CR49] Rosseel, Y. (2012). lavaan: An R package for structural equation modeling. *Journal of Statistical Software,**48*, 1–36. 10.18637/jss.v048.i02

[CR50] Rudman, L. A., & Phelan, J. E. (2008). Backlash effects for disconfirming gender stereotypes in organizations. *Research in Organizational Behavior,**28*, 61–79. 10.1016/j.riob.2008.04.003

[CR51] Sandberg, S. (2013). *Lean in: Women, work, and the will to lead*. Random House.

[CR52] Schmader, T., Johns, M., & Forbes, C. (2008). An integrated process model of stereotype threat effects on performance. *Psychological Review,**115*(2), 336–356. 10.1037/0033-295X.115.2.336 18426293 10.1037/0033-295X.115.2.336 PMC2570773

[CR53] Schmader, T., Bergsieker, H. B., & Hall, W. M. (2020). Cracking the culture code: A tri-level model for cultivating inclusion in organizations. In J. Forgas, B. Crano, & K. Fiedler (Eds.), *Applications of social psychology* (pp. 334–355). Taylor & Francis. 10.4324/9780367816407-17

[CR54] Seery, M. D. (2013). The biopsychosocial model of challenge and threat: Using the heart to measure the mind. *Social and Personality Psychology Compass,**7*, 637–653. 10.1111/spc3.12052

[CR55] Spencer, S. J., Logel, C., & Davies, P. G. (2016). Stereotype threat. *Annual Review of Psychology,**67*(1), 415–437. 10.1146/annurev-psych-073115-103235 26361054 10.1146/annurev-psych-073115-103235

[CR56] U.S. Bureau of Labor Statistics. (2023). *Employed persons by detailed occupation, sex, race, and Hispanic or Latino ethnicity*. U.S. Bureau of Labor Statistics. https://www.bls.gov/cps/cpsaat11.htm

[CR57] Vial, A. C., & Cimpian, A. (2024). Gender differences in children’s reasoning about and motivation to pursue leadership roles. *Sex Roles,**90*, 42–65. 10.1007/s11199-023-01428-z

[CR58] World Economic Forum. (2024). *Global gender gap report 2024*. https://www.weforum.org/publications/global-gender-gap-report-2024/

