# Supplementary material for: Emphasizing the Communal Demands of a Leader Role Makes Job Interviews Less Stressful for Women But Not More Successful
Source: Sex Roles. 2024 Oct 28;90(11):1506–20. doi: 10.1007/s11199-024-01509-7 (PMC11543713; doi:10.1007/s11199-024-01509-7)
Supplement: Supplementary file 1 — Supplementary file1 (DOCX 2035 KB) [file 11199_2024_1509_MOESM1_ESM.docx]

Online supplement for Nater, C., Eagly, A.H., Heilman, M.E., Messerli-Bürgy, N., & Sczesny, S. (2024). Emphasizing the communal demands of a leader role makes job interviews less stressful for women but not more successful. *Sex Roles*.Christa Nater, University of Bern. Email: [christa.nater@unibe.ch](mailto:christa.nater@unibe.ch)

**Contained within this document:**

**Supplement A:** Details of Sample in Hiring Simulation Study

**Supplement B:** List of Measures and Verbatim Research Materials

**Supplement C:** Vignette Study on Perceived Agentic and Communal Demands of Leader Role

**Supplement D:** Piecewise Growth Model Equations Examining Physiological Stress Responses

**Supplement E:** CFA and SEM Examining Effects of Gender on Evaluated Interview Success

**Additional Tables**

**Table S1:** Pearson Correlations, for Women (Lower Triangle) and Men (Upper Triangle) Separately

**Table S2:** Means, Standard Deviations, for Appraisal of Threat and Appraisal of Challenge Separately, by Participant Gender and Framing of Leader Role

**Table S3:** ANOVA Results for Appraisal of Threat and Appraisal of Challenge Separately, by Participant Gender and Framing of Leader Role

**Additional Figures**

**Figure S1:** Laboratory Procedure with the Cessation of the Stressor

**Supplement A: Details of Sample in Hiring Simulation Study**

All participants were management students at a large German-speaking University in central Europe. In terms of participants’ study level, 57% pursued a Bachelor’s degree, 42% pursued a Master’s degree, and 1% audited management classes without currently being enrolled in a degree program.

Did the female and male participants differ in the extent to which they internalized agentic and communal traits? During the habituation phase after participants arrived in the lab, they described themselves as agentic and communal on items defined by the assertiveness and compassion facets of the 60-item NEO Big Five Inventory (BFI-2; Danner et al., 2019; Soto & John, 2017). Both scales consisted of four items α for agency = .81, α for communion = .68. In line with gender stereotypes being internalized, women (*M* = 4.87, *SD* = 1.04) in the present sample reported having marginally lower agentic self-concepts than men (*M* = 5.15, *SD* = 1.05), *F*(1, 207) = 3.57, *p* = .060, η_p_^2^ = .017. Also, women (*M* = 5.85, *SD* = 0.78) reported having significantly higher communal self-concepts than men (*M* = 5.52, *SD* = 0.79), *F*(1, 207) = 9.23, *p* = .003, η_p_^2^ = .043.

**Supplement B: List of Measures and Verbatim Research Materials**

Below is the full list of variables included in the study. Variables analyzed and reported in the manuscript are denoted with an asterisk. The items of the variables are displayed thereafter.

- Demographics*
- Self-Esteem & Next Big Five Inventory (BFI-2) including Assertiveness*
- Perceived Fit for the Leader Role*
- Expected Performance in Job Interview*
- Self-evaluated Interview Success *
- Appraisal of Threat versus Challenge Experiences*
- Monitoring of Cues During the Job Interview
- Attractiveness of the Company
- Inclination to Apply
- Perceptions of Gender Bias
- Perception of Personnel Selection Committee

Thereafter, various questions assessed participants’ health, physical condition, sleeping habits, body mass index, medication consumption (including contraceptive method). These variables were assessed to bridge the time until the collection of the third and fourth salivary cortisol samples (+20min and +40min after the cessation of the job interview) and not analyzed. With the exemption of contraceptive method and body mass index, which were used as covariates in the cortisol analyses.

**Materials Used to Manipulate the Masculine vs. Feminine Emphasis of the Leader Role**


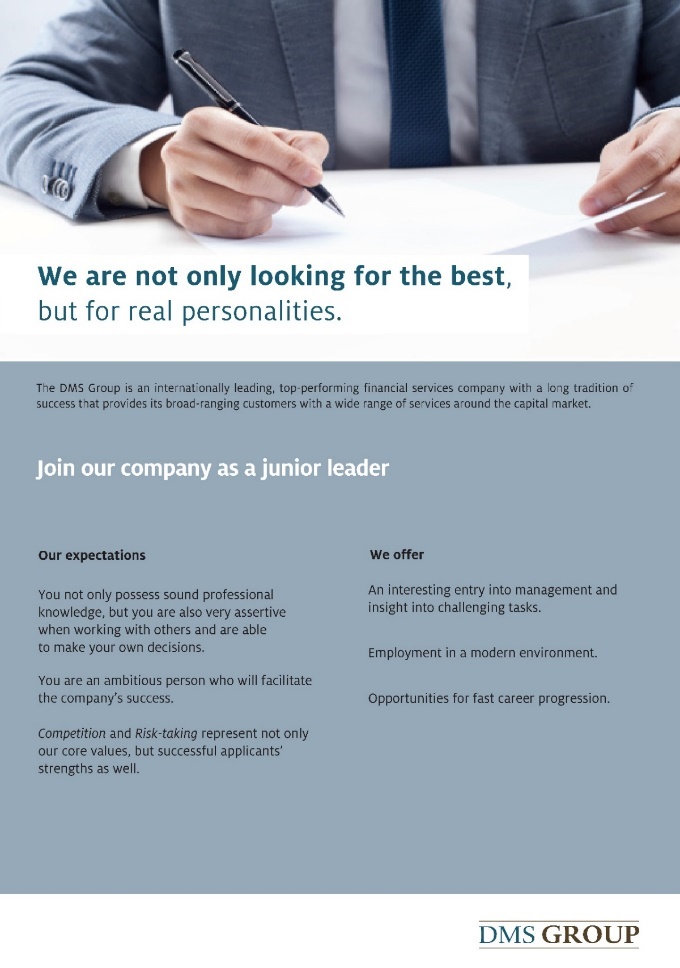

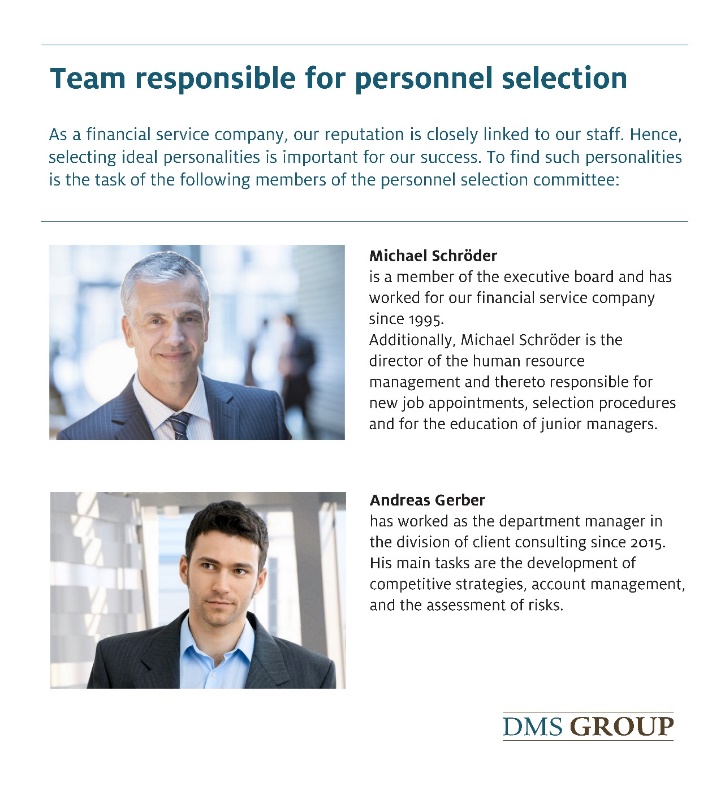

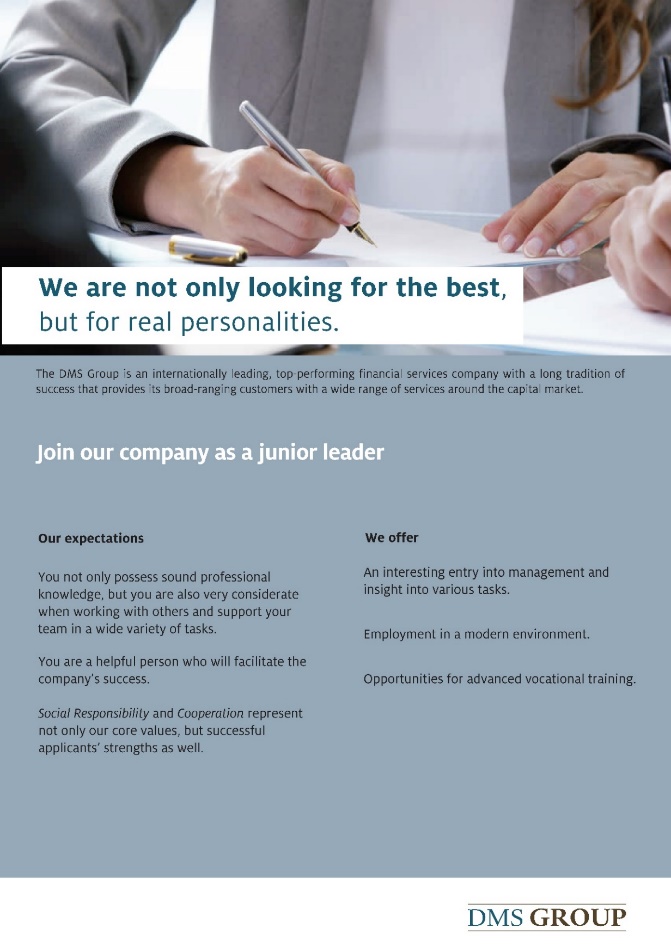

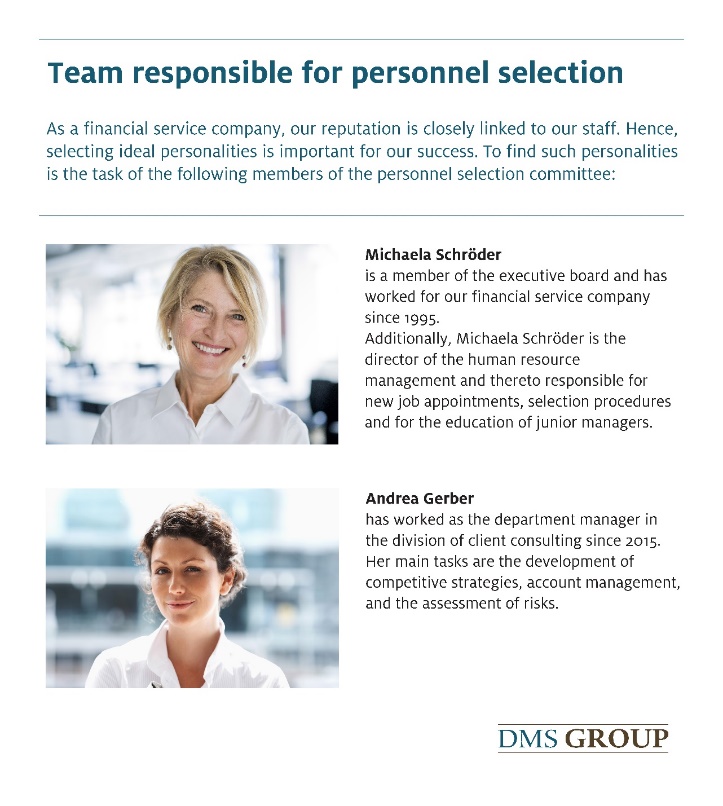


**Verbatim Questionnaire Administered in the Main Study**

**Demographics**

To begin with, please provide some demographic information for statistical purposes. Your anonymity is given at all times.

Gender?

- male
- female

Year of birth?

_ _ _ _

Country of origin? ____________________________

Country you live in? ____________________________

Are you currently doing a degree?

- Bachelor
- Master
- I do not study

Field of study? ____________________________

If you count all the semesters you have studied so far (Bachelor and Master), which semester are you currently in? ____________________________

Have you participated in job interviews before?

- yes, how many _____
- no

**QUESTIONAIRE PART 1**

**Self-esteem** (Rosenberg, 1965; von Collani & Herzberg, 2003)

*→ order was randomized*

Please read each statement carefully. Then indicate the extent to which you agree or disagree with each of the statements.

- On the whole, I am satisfied with myself.
- At times I think I am no good at all. (r)
- I feel that I have a number of good qualities.
- I am able to do things as well as most other people.
- I feel I do not have much to be proud of
- I certainly feel useless at times
- I feel that I'm a person of worth.
- I wish I could have more respect for myself. (r)
- All in all, I am inclined to think that I am a failure. (r)
- I take a positive attitude toward myself.

| Strongly disagree |  |  |  |  |  | Strongly agree |
| --- | --- | --- | --- | --- | --- | --- |
| ⃝ | ⃝ | ⃝ | ⃝ | ⃝ | ⃝ | ⃝ |

### Next Big Five Inventory (BFI-2) (Danner et al., 2019; Soto & John, 2017)

### *Facet Assertiveness was used (items marked with an *), the other facets were administered to hide the focus of present research*

### *→ order was randomized*

Here are a number of characteristics that may or may not apply to you. For example, do you agree that you are someone who likes to spend time with others? I am someone who . . .

For each of the following statements, please indicate to what extent you agree.

- Is outgoing, sociable
- Is compassionate, has a soft heart
- Tends to be disorganized (r)
- Is relaxed, handles stress well (r)
- Has few artistic interests (r)
- Has an assertive personality*
- Is respectful, treats others with respect
- Tends to be lazy (r)
- Stays optimistic after experiencing a setback (r)
- Is curious about many different things
- Rarely feels excited or eager (r)
- Tends to find fault with others (r)
- Is dependable, steady
- Is moody, has up and down mood swings
- Is inventive, finds clever ways to do things
- Tends to be quiet (r)
- Feels little sympathy for others (r)
- Is systematic, likes to keep things in order
- Can be tense
- Is fascinated by art, music, or literature
- Is dominant, acts as a leader*
- Starts arguments with others (r)
- Has difficulty getting started on tasks (r)
- Feels secure, comfortable with self (r)
- Avoids intellectual, philosophical discussions (r)
- Is less active than other people (r)
- Has a forgiving nature
- Can be somewhat careless (r)
- Is emotionally stable, not easily upset (r)
- Has little creativity (r)
- Is sometimes shy, introverted (r)
- Is helpful and unselfish with others
- Keeps things neat and tidy
- Worries a lot
- Values art and beauty
- Finds it hard to influence people (r)*****
- Is sometimes rude to others (r)
- Is efficient, gets things done
- Often feels sad
- Is complex, a deep thinker
- Is full of energy
- Is suspicious of others’ intentions(r)
- Is reliable, can always be counted on
- Keeps their emotions under control (r)
- Has difficulty imagining things (r)
- Is talkative
- Can be cold and uncaring (r)
- Leaves a mess, doesn’t clean up (r)
- Rarely feels anxious or afraid (r)
- Thinks poetry and plays are boring (r)
- Prefers to have others take charge (r)*
- Is polite, courteous to others
- Is persistent, works until the task is finished
- Tends to feel depressed, blue
- Has little interest in abstract Ideas (r)
- Shows a lot of Enthusiasm
- Assumes the best about people
- Sometimes behaves irresponsibly (r)
- Is temperamental, gets emotional easily
- Is original, comes up with new Ideas

| Strongly disagree |  |  |  |  |  | Strongly agree |
| --- | --- | --- | --- | --- | --- | --- |
| ⃝ | ⃝ | ⃝ | ⃝ | ⃝ | ⃝ | ⃝ |

You have now finished the first part of the questionnaire. Now, the main part of the study will begin, in which we examine how recruitment procedures are experienced and evaluated. To obtain valid and diagnostically conclusive results, we kindly ask you to imagine you were in a selection procedure for a job. Please take the job interview seriously, as if you were really looking for a job.

**Experimental Manipulation of Framing of Leadership Role**

*(→ random assignment)*

Now it’s your task to imagine being an applicant. You will next receive the job advertisement and the description of the personnel selection committee. The members of this committee will watch your video and rate your performance. They are trained to not only evaluate your spoken presentation but also your non-verbal behavior.

First, please read the job advertisement and the information about the committee carefully. Then, please respond to some open-ended questions in order to prepare for the interview. The time you have for each part will be indicated on the pages.

### Framing of Leader Role

- Job Advertisement (Timer was shown: 2 min, before it changed to next page, no “next” button)
- Personnel Selection Committee (Timer was shown: 1 min, before it changed to next page, no “next” button)

You now have 5 minutes to prepare for the job interview. As part of your preparation, please answer the questions below **using your own words**.

Then, please think freely about how you will handle and approach the job interview. Why are you the best candidate for the advertised position? What are your strengths and weaknesses regarding this position?

What kind of position is being advertised by the company?

________________________________________________________

________________________________________________________

What qualities and skills are expected in the ideal candidate?

________________________________________________________

________________________________________________________

**Perceived Fit for Leadership** (adapted from Sczesny & Bosak, 2008; Nater & Sczesny, 2016)

*→ order was randomized*

Before you proceed to the job interview, please respond to the following questions.

- I think that I am very well qualified for the advertised position.
- It will be easy for me to fulfill the job requirements.
- I meet the requirement profile for this position
- My skills and abilities are exactly appropriate for this position.

| Strongly disagree |  |  |  |  |  | Strongly agree |
| --- | --- | --- | --- | --- | --- | --- |
| ⃝ | ⃝ | ⃝ | ⃝ | ⃝ | ⃝ | ⃝ |

**Expected Performance in Job Interview** (Heilman, 1983, 2012)

I expect to perform well in this job interview.

| Strongly disagree |  |  |  |  |  | Strongly agree |
| --- | --- | --- | --- | --- | --- | --- |
| ⃝ | ⃝ | ⃝ | ⃝ | ⃝ | ⃝ | ⃝ |

**Job interview**

→ Participant was accompanied to the room next-door by male examiner

[*Instruction*] Please sit on the chair, I will give you a sign when you may begin. [*Start videotaping*]

You now have 5 minutes to present yourself as well as you can for the advertised position. Please look at camera. You may now begin.

In case the candidate stopped speaking before the 5 minutes were over, the interviewer waited 10 seconds and then asked the participant to continue. If the participant stopped speaking again, the interviewer asked questions that are commonly used in job interviews such as:

- What are your strengths and weaknesses regarding the advertised position?
- Why should you be chosen for this position?
- What distinguishes you from other applicants?
- Where do you see yourself in five years?
- Which aspects of a job are most important for you?

**QUESTIONAIRE PART 2**

**Self-evaluated Interview Success**

**Part 1: Subjective judgement** (adapted from Heilman et al., 1998)

How do you evaluate your own performance in the job interview for the leader position?

| incompetent | ⃝ | ⃝ | ⃝ | ⃝ | ⃝ | ⃝ | ⃝ | competent |
| --- | --- | --- | --- | --- | --- | --- | --- | --- |
| ineffective | ⃝ | ⃝ | ⃝ | ⃝ | ⃝ | ⃝ | ⃝ | effective |
| unconvincing | ⃝ | ⃝ | ⃝ | ⃝ | ⃝ | ⃝ | ⃝ | convincing |
| disorganized | ⃝ | ⃝ | ⃝ | ⃝ | ⃝ | ⃝ | ⃝ | organized |
| indecisive | ⃝ | ⃝ | ⃝ | ⃝ | ⃝ | ⃝ | ⃝ | decisive |
| insecure | ⃝ | ⃝ | ⃝ | ⃝ | ⃝ | ⃝ | ⃝ | confident |

Overall, how well or poorly do you evaluate your own performance in the job interview?

| Very poorly |  |  |  |  |  | Very well |
| --- | --- | --- | --- | --- | --- | --- |
| ⃝ | ⃝ | ⃝ | ⃝ | ⃝ | ⃝ | ⃝ |

**Part 2: Common rule scale** (self-developed)

Please evaluate your performance compared to 100 applicants who also did the interview and presented themselves:

- Where do you think you would rank your performance?
- How convincingly did you present yourself as the ideal person for the position?
- How good was the impression you made as the ideal person for the position?
- How successfully did you present yourself as meeting the requirements for the position?

| Worst performance |  |  |  | Average |  |  |  | Best performance |
| --- | --- | --- | --- | --- | --- | --- | --- | --- |


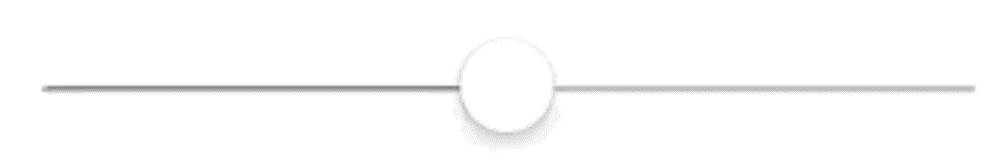


50

**Part 3: Subjective judgement** (self-developed)

How likely do you think it is that you would make it to the short list?

| Very unlikely |  |  |  |  |  | Very likely |
| --- | --- | --- | --- | --- | --- | --- |
| ⃝ | ⃝ | ⃝ | ⃝ | ⃝ | ⃝ | ⃝ |

**• Appraisal of Threat versus Challenge** (adapted from PASA; Gaab, 2009) *→ order was randomized*

** marks items from the threat subscale*

*^#^ marks items from the challenge subscale*

Now please answer some questions about your job interview.

- I felt threatened by the situation.*
- The situation was relevant for me.^#^
- In this situation, I knew what to do.
- It depended mainly on me whether I could handle the situation.
- The situation was very uncomfortable for me.*
- The situation had no effect on me. (r) ^#^
- I had no idea what to do. (r)
- It was above all my own behavior that allowed me to protect myself from failure in this situation.
- I was worried, because the situation was threatening to me.*
- The situation was not a challenge for me. (r) ^#^
- In the situation, many options for action occurred to me.
- I had control over many things that happened in this situation.
- The situation did not frighten me. (r)*
- The situation challenged me. ^#^
- Many solutions for this situation occurred to me.
- Having mastered this situation is due to my efforts and my commitment.
- I was able to cope well with this situation.

| Not at all |  |  |  |  |  | Very much |
| --- | --- | --- | --- | --- | --- | --- |
| ⃝ | ⃝ | ⃝ | ⃝ | ⃝ | ⃝ | ⃝ |

### Monitoring of Cues During the Job Interview (Verbal thought questionnaire; Beilock, Rydell, & McConnell, 2007)

We all have several thoughts that run through our mind at any given time. Please describe everything that you remember thinking about as you presented yourself in the job interview.

________________________________________________________

________________________________________________________

________________________________________________________

(self-developed): Please indicate whether the following thoughts ran through your mind during the job interview. I worried about…

- not seeming assertive and competitive.*
- not seeming competent and confident.*
- looking unqualified.*
- seeming passive and tentative.*

| Not at all |  |  |  |  |  | Very much |
| --- | --- | --- | --- | --- | --- | --- |
| ⃝ | ⃝ | ⃝ | ⃝ | ⃝ | ⃝ | ⃝ |
|  |  |  |  |  |  |  |
|  |  |  |  |  |  |  |

**Additional variables**

### Attractiveness of the Company

- I think I would like to work at a place like DMS Group.
- I think I would like to work in a company that has similar hiring practices as those of DMS Group.
- I think I would like to work under the supervision of people with similar values as the staff.
- I think I could “be myself” at a company like DMS Group
- I think I would be willing to put in extra effort if my supervisor asked me to.
- I think my colleagues at DMS Group would become my close personal friends.
- I think I would be willing to put in a great deal of effort beyond that normally expected in order to help DMS Group be successful.
- I think I would be treated fairly by my supervisor.
- I think I would trust the management to treat me fairly.
- I think that my values and the values of DMS Group are very similar.
- I think that the DMS Group environment would inspire me to do the very best job that I can.

| Disagree |  |  |  |  |  | Agree |
| --- | --- | --- | --- | --- | --- | --- |
| ⃝ | ⃝ | ⃝ | ⃝ | ⃝ | ⃝ | ⃝ |

**Inclination to Apply**

If you would have already finished your studies: What do you think, would you actually apply for this job?

| Not at all |  |  |  |  |  | Very much |
| --- | --- | --- | --- | --- | --- | --- |
| ⃝ | ⃝ | ⃝ | ⃝ | ⃝ | ⃝ | ⃝ |

### Perceptions of Gender Bias (adapted from Bergeron, Block, & Echtenkamp, 2006)

- The recruiter expected me to do poorly on the job interview because of my gender.
- I doubt that others would think that I have less leadership ability because of my gender. (r)
- Some people feel I have less leadership ability because of my gender.
- People of my gender rarely face unfair evaluations in job interviews. (r)
- In job interviews, people of my gender often face biased evaluations from others.
- My gender does not affect people’s perception of my leadership ability. (r)
- In job interviews, others may look down on me because of my gender.

| Strongly disagree |  |  |  |  |  | Strongly agree |
| --- | --- | --- | --- | --- | --- | --- |
| ⃝ | ⃝ | ⃝ | ⃝ | ⃝ | ⃝ | ⃝ |

**Closing Remarks by participants**

If you wish to make any remark about the study, please use the space below.

________________________________________________________

________________________________________________________

________________________________________________________

________________________________________________________

________________________________________________________

What do you think was the purpose or aim of the present research?

________________________________________________________

________________________________________________________

________________________________________________________

________________________________________________________

________________________________________________________

**Perception of Personnel Selection Committee**

The personnel selection committee seemed…

|  | not at all |  |  |  |  |  | very |
| --- | --- | --- | --- | --- | --- | --- | --- |
| competent | ⃝ | ⃝ | ⃝ | ⃝ | ⃝ | ⃝ | ⃝ |
| likeable | ⃝ | ⃝ | ⃝ | ⃝ | ⃝ | ⃝ | ⃝ |
| attractive | ⃝ | ⃝ | ⃝ | ⃝ | ⃝ | ⃝ | ⃝ |

**QUESTIONNAIRE PART 3:**

**Covariates for Cortisol and Heart Rate Variability**

Some information is needed for the valid measurement of psychophysiological stress reactions. At the end, please answer a few questions about your **current health**, **daily stress**, **physical condition**, **sleeping habits**, **tobacco**, **alcohol** and **medication consumption**.

**Current health**

Have you been ill in the last 2-3 days? If so, what did you suffer from?

- No
- Yes: ______________________

**PHQ-D**: #1 Level of the impairment due to discomfort (Löwe et al., 2002)

How much did you feel affected by the following symptoms during the last 4 weeks?

|  | Unaffected | Slightly affected | Strongly affected |
| --- | --- | --- | --- |
| Stomach pains | ◌ | ◌ | ◌ |
| Backaches | ◌ | ◌ | ◌ |
| Pain in arms, legs or joints (knees, hips, etc.) | ◌ | ◌ | ◌ |
| Menstrual pain or other problems related to menstruation* | ◌ | ◌ | ◌ |
| Pain or problems during sexual intercourse | ◌ | ◌ | ◌ |
| Headaches | ◌ | ◌ | ◌ |
| Chest pain | ◌ | ◌ | ◌ |
| Dizziness | ◌ | ◌ | ◌ |
| Faint spells | ◌ | ◌ | ◌ |
| Palpitations | ◌ | ◌ | ◌ |
| Shortness of breath | ◌ | ◌ | ◌ |
| Constipation, nervous bowel, or diarrhea | ◌ | ◌ | ◌ |
| Nausea, flatulence, or digestive problems | ◌ | ◌ | ◌ |

* for women only

**PHQ-D**: #5 Frequency of nervousness, anxiety, etc. (Löwe et al., 2002)

How often did you feel affected by the following symptoms during the last 4 weeks?

|  | Not at all | On some days | On more than half of the days |
| --- | --- | --- | --- |
| Nervousness, anxiety, tension or excessive concern | ◌ | ◌ | ◌ |
| Feelings of restlessness, making it difficult to sit still | ◌ | ◌ | ◌ |
| Easy to fatigue | ◌ | ◌ | ◌ |
| Muscle tension, muscle pain | ◌ | ◌ | ◌ |
| Difficulty falling asleep or sleeping through the night | ◌ | ◌ | ◌ |
| Difficulty concentrating on something, e.g. reading or watching television | ◌ | ◌ | ◌ |
| Slight irritability, hypersensitivity | ◌ | ◌ | ◌ |
|  |  |  |  |

**PHQ-D**: #2 Frequency of impairment due to discomfort (Löwe et al., 2002)

How often did you feel affected by the following symptoms during the last 2 weeks?

|  | Not at all | On some days | On more than half of the days | Almost every day |
| --- | --- | --- | --- | --- |
| Little interest or pleasure in your activities | ◌ | ◌ | ◌ | ◌ |
| Depression, melancholy or hopelessness | ◌ | ◌ | ◌ | ◌ |
| Trouble falling asleep or sleeping through, or increased sleep | ◌ | ◌ | ◌ | ◌ |
| Tiredness or feeling of not having any energy | ◌ | ◌ | ◌ | ◌ |
| Decreased appetite or excessive need to eat | ◌ | ◌ | ◌ | ◌ |
| Bad opinion of oneself; Feeling of being a failure or having disappointed family | ◌ | ◌ | ◌ | ◌ |
| Difficulty concentrating on something, e.g. reading a newspaper or watching television | ◌ | ◌ | ◌ | ◌ |
| Were your movements or your language so slow that others would notice? Or, on the contrary, were you «fidgety» or restless and thus had a stronger urge to move than usual? | ◌ | ◌ | ◌ | ◌ |
| Thoughts that you would rather be dead or want to inflict suffering on yourself | ◌ | ◌ | ◌ | ◌ |

Dimension, similar to the concept “Burnout”

How often have you made the following experiences during the last 4 weeks?

|  | Not at all | On some days | On more  than half of the days | Almost every day |  |
| --- | --- | --- | --- | --- | --- |
| Fear of something unpleasant happening. | ◌ | ◌ | ◌ | ◌ |  |
| I try in vain to get recognition for good performance. | ◌ | ◌ | ◌ | ◌ |  |
| Times when I have too many obligations to fulfil. | ◌ | ◌ | ◌ | ◌ |  |
| Times when I cannot suppress worried thoughts. | ◌ | ◌ | ◌ | ◌ |  |
| Although I do my best, my work is not appreciated. | ◌ | ◌ | ◌ | ◌ |  |
| The experience that everything I have to do is too much. | ◌ | ◌ | ◌ | ◌ |  |
| Times when I worry a lot and can’t stop. | ◌ | ◌ | ◌ | ◌ |  |
| Times when I don’t deliver the performance expected of me. | ◌ | ◌ | ◌ | ◌ |  |
| Times when responsibility for others become a burden. | ◌ | ◌ | ◌ | ◌ |  |
| Times when my work grows beyond my head. | ◌ | ◌ | ◌ | ◌ |  |
| Fear of not being able to fulfil my tasks. | ◌ | ◌ | ◌ | ◌ |  |
| Times when my worries are over my head. | ◌ | ◌ | ◌ | ◌ |  |

**PHQ-D**: #12 impairment due to discomfort (Löwe et al., 2002)

How much did you feel affected by the following symptoms during the last 4 weeks?

|  | Unaffected | Slightly affected | Strongly affected |
| --- | --- | --- | --- |
| Worries about your health | ◌ | ◌ | ◌ |
| Worries about your weight or appearance | ◌ | ◌ | ◌ |
| Little or no sexual desire or pleasure during sexual intercourse | ◌ | ◌ | ◌ |
| Difficulties with spouse, partner, girlfriend/boyfriend | ◌ | ◌ | ◌ |
| Burden of caring for children, parents or other family members | ◌ | ◌ | ◌ |
| Stress at work or during studies | ◌ | ◌ | ◌ |
| Financial problems or worries | ◌ | ◌ | ◌ |
| Not to have anyone to talk to about problems. | ◌ | ◌ | ◌ |
| Something bad happened recently | ◌ | ◌ | ◌ |
| Thoughts or dreams of terrible events in the past – e.g. the destruction of one’s own home, a serious accident, physical violence or a sexual act under coercion | ◌ | ◌ | ◌ |

**Important live events** (adapted from *Coddington live events scale*; Coddington, 1972; PHQ-D: #13 Gewalt, Löwe et al., 2002)

With the following questions we would like to find out whether you have suffered a severe stroke of fate in the last 12 months. In particular, we would like to know how much this life event has stressed you.

|  |  | No stress |  |  | A lot of stress |
| --- | --- | --- | --- | --- | --- |
|  | Death of a relative | ◌ | ◌ | ◌ | ◌ |
|  | Severe disease | ◌ | ◌ | ◌ | ◌ |
|  | Chronic illness | ◌ | ◌ | ◌ | ◌ |
|  | Serious accident | ◌ | ◌ | ◌ | ◌ |
|  | Victims of violence | ◌ | ◌ | ◌ | ◌ |
|  | Victim of abuse | ◌ | ◌ | ◌ | ◌ |
|  | Coercion to unwanted sexual acts | ◌ | ◌ | ◌ | ◌ |
|  | Unemployment | ◌ | ◌ | ◌ | ◌ |
|  | Invalidity | ◌ | ◌ | ◌ | ◌ |
|  | Alcohol/drugs (also cannabis) | ◌ | ◌ | ◌ | ◌ |
|  | Newborn in the family during the last 12 months | ◌ | ◌ | ◌ | ◌ |
|  | Loss of a pet in the last 12 months | ◌ | ◌ | ◌ | ◌ |
|  | Divorce or separation | ◌ | ◌ | ◌ | ◌ |
|  | House moving | ◌ | ◌ | ◌ | ◌ |
|  | Other, namely: …………………… | ◌ | ◌ | ◌ | ◌ |
|  | Other, namely: …………………… | ◌ | ◌ | ◌ | ◌ |
|  | Other, namely: …………………… | ◌ | ◌ | ◌ | ◌ |

**Important life events: Daily problems** (adapted from *Coddington live events scale*; Coddington, 1972)

Which problems have you been confronted with in the last 12 months?

|  |  | No stress |  |  | A lot of stress |
| --- | --- | --- | --- | --- | --- |
|  | Problems during studies | ◌ | ◌ | ◌ | ◌ |
|  | Problems with your partner | ◌ | ◌ | ◌ | ◌ |
|  | Problems with other people (family, friends) | ◌ | ◌ | ◌ | ◌ |
|  | Problems at work | ◌ | ◌ | ◌ | ◌ |
|  | Other, namely: …………………… | ◌ | ◌ | ◌ | ◌ |
|  | Other, namely: …………………… | ◌ | ◌ | ◌ | ◌ |
|  | Other, namely: …………………… | ◌ | ◌ | ◌ | ◌ |

**PHQ-D:** #14 Burdens (Löwe et al., 2002)

What burdens you most in your life at the moment?

_______________________________________

**Tobacco consume**

**The following questions relate to tobacco consume.**

Do you smoke, even if only seldom?

◌ yes ◌ no

If yes, how many cigarettes do you smoke on average **per day**?

___ Cigarettes

If you smoke less than 1 cigarette a day, how many cigarettes do you smoke on average **per week**?

___ Cigarettes

When do you smoke your first cigarette after getting up?

◌ Within 5 minutes

◌ Within 6 to 30 minutes

◌ Within 31 to 60 minutes

◌ It takes longer than 60 minutes

Do you find it hard to refrain from smoking in places where smoking is prohibited (e.g. library, cinema)?

◌ yes ◌ no

Which cigarette would you not want to renounce?

◌ The first after getting up

◌ Another one

Do you generally smoke more during the first hours after awakening than during the rest of the day?

◌ yes ◌ no

Does it happen that you smoke when you are ill and have to stay in bed during the day?

◌ yes ◌ no

**PHQ-D**: #9, 10 Alcohol consumption (Löwe et al., 2002)

Do you sometimes drink alcohol (including beer or wine)?

◌ no ◌ yes

Have you experienced any of the following situations more than once in the last 6 months?

|  | No | Yes |
| --- | --- | --- |
| You have been drinking alcohol even though a doctor advised you to stop drinking for health reasons? | ◌ | ◌ |
| Have you drunk alcohol, been drunk or "hungover" at work, at school, while caring for children or in fulfilling other obligations? | ◌ | ◌ |
| Have you been away from work, school or other obligations or have you been late because you have been drinking or "hungover"? | ◌ | ◌ |
| You had difficulty getting along with others because you had been drinking? | ◌ | ◌ |
| You drove a car after drinking several glasses or too much of alcohol? | ◌ | ◌ |
|  |  |  |

**Moderate-to-vigorous activity**

The next questions relate to the time you have been physically active during the **last 7 days**. Please remember the activities you do at work, in your home and garden, to get from one place to another, and in your free time for recreation, training and sports.

Think of all your **intense physical activities** over the **past 7 days**. Heavy or intense activities are activities that require a lot of physical exertion, and during which you breathe significantly more than normal. Think *only* of the physical activities you have done for **at least 10 minutes without interruption**.

- On how many of the **past 7 days** have you been doing intensive physical activity such as heavy lifting, digging, aerobics or fast cycling?

___  **Days per week**

◌ No intense physical activities

- How much **time** did you usually spend on one of these days with intensive physical activities (e.g. 1.5 h = 1 hour per day + 30 minutes per day or 0.5 h = 0 hours per day + 30 minutes per day)?

___  **Hours per day**

___  **Minutes per day**

◌ I do not know / I am unsure

**Sleeping habits**

The following questions concern your **sleeping habits**.

- When did you usually go to bed in the evening during the last four weeks?

Usual time: _______

- How long did it usually take for you to fall asleep at night during the last four weeks?

in minutes: _______

- When did you usually get up in the morning during the last four weeks?
  Usual time: _______
- How many hours per night have you actually slept during the last four weeks? (Doesn't have to match the number of hours you spent in bed)

Effective sleep time (hours): _______

- Do you have a part-time job that involves shift work?

🔿 no 🔿 yes

- How many nights a week do you do a night shift there? _______
- When was your last night shift? _______days ago

For each of the following questions, please tick the answer that applies to you. Please answer all questions.

How often have you slept badly during the last four weeks?

- because you couldn't fall asleep within 30 minutes?
- because you woke up in the middle of the night or early in the morning?
- because you had to get up to go to the toilet?
- because you had trouble breathing?
- because you had to cough or snore out loud?
- because you were too cold?
- because you were too warm?
- because you had a bad dream?
- because you were in pain?
- for other reasons? Which ones?

m Not at all in the last four weeks

m Less than once a week

m Once or twice a week

m Three or more times a week

**PHQ-D:** #16 Menstrual flow (Löwe et al., 2002)

* for women only

What is the best way to describe your menstruation?

◌ Menstruation is unchanged

◌ No menstruation due to pregnancy or recent childbirth

◌ Menstruation has become irregular or duration, distance or strength have changed

◌ No menstruation for at least one year

◌ Menstruation with hormone therapy (taking oestrogens) or contraception with the pill

|  | No / Does not apply | Yes |
| --- | --- | --- |
| In the week before your menstrual period begins, do you have any pronounced problems with your mood - e.g. depression, anxiety, irritability, aggressiveness or mood swings? | ◌ | ◌ |
| Do these problems disappear at the end of your menstrual period? | ◌ | ◌ |
| Have you given birth in the last 6 months? | ◌ | ◌ |
| Have you had a miscarriage during the last 6 months? | ◌ | ◌ |
| Are you having trouble getting pregnant? | ◌ | ◌ |

**Time of menstruation**

1. Do you have a regular menstrual cycle (period)?

🔿 yes 🔿 no

1. When as the first day of your last period? Please indicate the date as accurately as possible.

_________

1. How many days does your menstrual period usually last? _____ days
2. Which contraceptive method do you use? __________

**PHQ-D**: #14 Medications (Löwe et al., 2002)

Do you regularly take medication?

◌ no ◌ yes

What **medications** (**name**) are you taking? ___________

**Body Mass Index**

How tall are you? ____ cm

How much do you weight? _____ kg

**Socio-economic status of parents ISEI** (Ganzeboom et al., 1992) coded based on International Standard Classification of Occupations ISCO-08 (International Labour Office, 2012)

What is your father's profession?

______________________

What is your mother's profession?

______________________

**Thank you for your participation** which is highly appreciated. Please wait until the examiner comes to see you. In the meantime, feel free to read the magazines provided on the desk.

**Supplement C**: Vignette Study on Perceived Agentic and Communal Demands of Leader Role

**Method**

**Participants and Design**

An *a priori* power analysis based on the framing effect on perceived fit found in the main study (*η_p_^2^* = 0.031) indicated the need of at least 246 participants (1-*β* = 0.80) to detect a main effect of leader role framing. After the exclusion of 11 participants who failed to correctly indicate that the job was a leadership position, the final sample consisted of 305 participants (189 women, 111 men, five diverse). Participants ranged in age from 19 to 40 years (*M* = 24.30, *SD* = 3.23). These students were enrolled in the same university as participants in the main study but had not taken part in the hiring simulation study.

**Procedure and Materials**

This online study presented participants with either the masculine- or feminine-framed leader role used in the main study. After learning about the role, participants indicated the extent to which good performance in the position required agentic qualities and communal qualities. Five-items assessed perceived agentic qualities (e.g., being decisive, determined) and five items communal qualities (e.g., being cooperative, other-oriented), on 7-point rating scales that ranged from (1) *not at all* to (7) *very much*. Both resulting scales had acceptable internal consistency, α = .76 for agency, and α = .93 for communion.

**Transparency and Openness**

The data and analysis code are available on OSF (<https://osf.io/pefc9>).

**Results and Discussion**

Supplement Table C1 displays the means and standard deviations. One sample *t*-tests examined whether the mean levels for required agency and required communion in the two roles were significantly above the scale midpoint. As expected, results showed that agentic requirements were significantly above the midpoint in both the masculine framing (*M* = 6.17, *SD* = 0.72), *t*(150) = 36.97, *p* < .001, and the feminine framing (*M* = 5.49, *SD* = 0.85), *t*(153) = 21.88, *p* < .001. For communion, however, mean levels were above the midpoint in the feminine condition (*M* = 5.92, *SD* = 1.10), *t*(153) *=* 21.72, *p* < .001, but not the masculine condition (*M* = 3.94, *SD* = 1.39), *t*(150) = -0.53, *p* = 0.599.

Additional ANOVAs were conducted on the two gender-neutral competence items that were used as fillers to mask the focus of the study on agentic and communal demands. Results found no effect of the framing condition on intelligent and organized: Participants indicated that high levels of intelligence were required for both the feminine (*M* = 5.03, *SD* = 1.36) and masculine (*M* = 5.25, *SD* = 1.29) framed role, *F*(1, 303) = 1.95, *p* = .163, *η_p_^2^* = .006. Also, high levels of being organized was demanded in both the feminine (*M* = 5.58, *SD* = 1.19) and masculine (*M* = 5.44, *SD* = 1.20) framed role, *F*(1, 303) = 1.06, *p* = .305, *η_p_^2^* = .003. In sum, both leader roles were perceived to have high levels of competence demands.

**Supplement Table C1**

*Means and Standard Deviations for Perceived Agentic and Communal Demands, by Framing of Leader Role (Feminine vs. Masculine)*

|  | |  | Perceived communal  demands | |  | Perceived agentic demands | |  |
| --- | --- | --- | --- | --- | --- | --- | --- | --- |
|  |  |  |  |  |  |  |  |  |
| Experimental design | *N** |  | *M* | *SD* |  | *M* | *SD* |  |
| Feminine | 154 |  | 5.92 | 1.10 |  | 5.49 | 0.85 |  |
| Masculine | 151 |  | 3.94 | 1.39 |  | 6.17 | 0.72 |  |

*Note*. The scales for required communal and agentic qualities
ranged from (1) *not at all* (7) *very much*.

**Verbatim Study Materials of Online Vignette Study**

**Introduction**

Dear students,

In this study, we investigate whether job advertisements can provide evidence of behaviors and qualities that most likely lead to a good performance evaluation in the position.

This survey will take 5 minutes. There are no right or wrong answers, we are interested in your personal opinions.

Thank you for your valuable participation.

**Framing of Leadership Role as used in Main Study**

*(→ random assignment)*

Please read the job advertisement on the next page carefully. Please think about what is required for a **good performance evaluation** in this **leadership role** after 6 months.

A) Feminine framing of leadership role

B) Masculine framing of leadership role

**Items**

*(→ randomized)*

*Asterisks denote the items of the agency scale and † the items of the communion scale

Please indicate what you think is required for a **good performance evaluation** in this **leadership position**.

| \|  \| Not at all \|  \|  \|  \|  \|  \| Very much \| \| --- \| --- \| --- \| --- \| --- \| --- \| --- \| --- \| \| decisive* \| ⃝ \| ⃝ \| ⃝ \| ⃝ \| ⃝ \| ⃝ \| ⃝ \| \| determined* \| ⃝ \| ⃝ \| ⃝ \| ⃝ \| ⃝ \| ⃝ \| ⃝ \| \| persuasive* \| ⃝ \| ⃝ \| ⃝ \| ⃝ \| ⃝ \| ⃝ \| ⃝ \| \| task-oriented* \| ⃝ \| ⃝ \| ⃝ \| ⃝ \| ⃝ \| ⃝ \| ⃝ \| \| cooperative† \| ⃝ \| ⃝ \| ⃝ \| ⃝ \| ⃝ \| ⃝ \| ⃝ \| \| supportive† \| ⃝ \| ⃝ \| ⃝ \| ⃝ \| ⃝ \| ⃝ \| ⃝ \| \| understanding† \| ⃝ \| ⃝ \| ⃝ \| ⃝ \| ⃝ \| ⃝ \| ⃝ \| \| other-oriented† \| ⃝ \| ⃝ \| ⃝ \| ⃝ \| ⃝ \| ⃝ \| ⃝ \| \| diligent \| ⃝ \| ⃝ \| ⃝ \| ⃝ \| ⃝ \| ⃝ \| ⃝ \| \| intelligent \| ⃝ \| ⃝ \| ⃝ \| ⃝ \| ⃝ \| ⃝ \| ⃝ \| \| organized \| ⃝ \| ⃝ \| ⃝ \| ⃝ \| ⃝ \| ⃝ \| ⃝ \| \| resilient \| ⃝ \| ⃝ \| ⃝ \| ⃝ \| ⃝ \| ⃝ \| ⃝ \| |
| --- | --- | --- | --- | --- | --- | --- | --- | --- | --- | --- | --- | --- | --- | --- | --- | --- | --- | --- | --- | --- | --- | --- | --- | --- | --- | --- | --- | --- | --- | --- | --- | --- | --- | --- | --- | --- | --- | --- | --- | --- | --- | --- | --- | --- | --- | --- | --- | --- | --- | --- | --- | --- | --- | --- | --- | --- | --- | --- | --- | --- | --- | --- | --- | --- | --- | --- | --- | --- | --- | --- | --- | --- | --- | --- | --- | --- | --- | --- | --- | --- | --- | --- | --- | --- | --- | --- | --- | --- | --- | --- | --- | --- | --- | --- | --- | --- | --- | --- | --- | --- | --- | --- | --- | --- |
|  |

To what extent do you think the **junior leader position** requires behavior or qualities that are relevant for…

* behaving competitively and assertively in order to pursue and achieve the goals of the company.

| Not at all |  |  |  |  |  | Very much |
| --- | --- | --- | --- | --- | --- | --- |
| ⃝ | ⃝ | ⃝ | ⃝ | ⃝ | ⃝ | ⃝ |

† establishing and maintaining social relationships and caring about good social functioning within the company.

| Not at all |  |  |  |  |  | Very much |
| --- | --- | --- | --- | --- | --- | --- |
| ⃝ | ⃝ | ⃝ | ⃝ | ⃝ | ⃝ | ⃝ |

**Manipulation Check**

Finally, we are interested to learn what information you remember from the job advertisement.

Which position was advertised in the job ad?

◌ Elementary School Teaching Position

◌ Junior Leadership Position

◌ Senior Electrician position

◌ I don’t remember

**Demographics**

Please provide us with some demographic information. Your anonymity is given at all times.

Gender?

◌ Woman

◌ Man

◌ other: ___________

Year of birth? _ _ _ _

Are you currently studying?

◌ Bachelor

◌ Master

◌ I do not study.

Field of study? ______________

Nationality? ______________

**Remarks**

If you wish to make any comments about the study, please use the space below. Thank you for your participation, which we highly appreciate.

**Supplement D: Piecewise Growth Model Equations Examining Physiological Stress Responses**

The interaction between gender, framing, and each of the two-phase variables (i.e., Framing × Gender × Phase 1, for reactivity; Framing × Gender × Phase 2, for recovery) represents the effect of the interaction between gender and framing on the slope of cortisol over the study. The model equation for cortisol release in each participant can be represented as:

$$y_{ij}=\beta_{0j}+\beta_{1j}{Phase1}_{ij}+\beta_{2j}{Phase2}_{ij}+e_{ij}$$

The variation of the regression coefficients 𝛽_j_ is modeled by a person-level regression model:


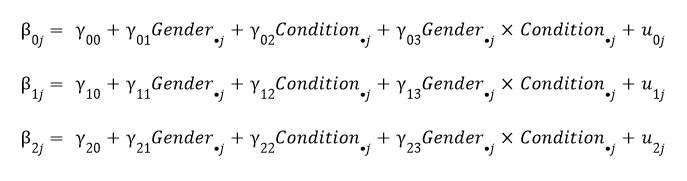


Furthermore, in order to control for the impact of BMI and women’s use of birth control may have on cortisol levels, we reran the model twice and added each variable to the between-subjects level of the MLM model according to the following equation (example for BMI):

$$y_{ij}=\beta_{0j}+\beta_{1j}{Phase1}_{ij}+\beta_{2j}{Phase2}_{ij}+e_{ij}$$

The variation of the regression coefficients 𝛽_j_ is modeled by a person-level regression model:


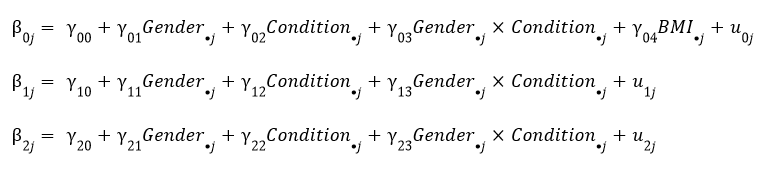


**Results for Stress Recovery (Phase 2), Modeled by the t3 and t4 Measures**

As shown in Table 5 in the main manuscript, for recovery, the Gender × Phase 2 interaction was significant and the Framing × Phase 2 and the Framing × Gender × Phase 2 interactions were nonsignificant. Decomposition of the significant Gender × Phase 2 interaction within levels of gender found a significant negative slope for men, *B* = -0.20, *SE* = 0.06, *df* = 384.75, *t* = -3.58, *p* < .001, but not women, *B* = -0.04, *SE* = 0.05, *df* = 384.75, *t* = -0.92, *p* = .361, indicating that men’s cortisol levels decreased more than women’s did. Taken together, results on physiological stress recovery suggest that after the interview men’s stress levels lowered to a greater extent than women’s, although the framing did not differently affect women’s and men’s recovery.

**Supplement E: CFA and SEM Examining Effects of Gender on Evaluated Interview Success**

**Supplement Table E1**

*Confirmatory Factor Analysis to Ensure One-factor Models for Measures Included in the SEM*

| Measure | *χ^2^* | *df* | CFI | TLI | RMSEA [90% CI] |
| --- | --- | --- | --- | --- | --- |
| Self-ascribed agency | 4.02 | 2 | .992 | .977 | .069 [.001, .169] |
| Perceived fit | 4.55 | 2 | .996 | .989 | .078 [.001, .173] |
| Expected performance | − | − | − | − | − |
| Ratio threat and challenge appraisal | − | − | − | − | − |
| Self-evaluated interview success | 150.70* | 54 | .961 | .952 | .093 [.075, .110] |

*Note*. CFI = comparative fit index; TLI = Tucker-Lewis index; RMSEA = root-mean-square error of approximation; CI = confidence interval. No CFA was performed for expected performance (measured with a single item) and ratio of threat and challenge appraisal. Self-ascribed agency was only included in the addition model presented in Figure E2 below.

* *p* < .05

**PART 1: Moderated Mediation Model: Testing Moderation by Framing of Leadership Role**

A multigroup structural equation analysis tested whether the structural paths from participant gender to perceived fit, expected performance for the job interview, appraisal of threat versus challenge, and to the two evaluations of interview success differed for the feminine and masculine framing of the leadership role (i.e., moderation analysis).

In sum, given the presence of measurement invariance across the two framings of the roles (see next section), we constrained the structural paths to be equal between the feminine and masculine condition and tested the significance of the *χ^2^* change. Results indicated that these restrictions across the conditions did not significantly reduce the overall model fit compared to the unrestricted model, Δ*χ^2^* = 0.823, Δ*df*= 1, *p* = .364, indicating that framing of the leadership role did not moderate the structural paths in the proposed model. We next present the results for each step separately.

**Measurement Invariance Analysis Across the Two Framings of the Leader Role**

Prior to the test of whether the structural paths from participant gender through perceived fit, expected performance in job interview, subjective stress, to self-evaluations would be different for the leader role with a masculine and feminine framing (i.e., moderation analysis), we tested the assumptions of configurable invariance, metric measurement invariance, and scalar measurement invariance (e.g., Milfont & Fischer, 2010). These assumptions are typically evaluated by comparing the fit of two consecutive, nested models that are identical except for a target set of restrictions in one of the two models (Putnick & Bornstein, 2016). Measurement noninvariance would suggest that a construct has a different structure or meaning to the feminine and masculine framing of the leadership role, and that therefore the construct cannot be meaningfully tested or construed across the conditions.

**Configural Invariance**

Configural invariance requires that a given set of items is predicted by the same latent variable with the same pattern of factor loadings. Therefore, the model with separate parameters for the masculine and feminine framing was estimated (i.e., with twice as many model parameters). The overall model fit thus informed about the cross-framing condition invariance with respect to the configural structure, that is, whether the factor model with the given factors and loadings is invariant across the two conditions. Results showed that all items loaded significantly on the same factors in both conditions and that the model fit was comparable in both framing conditions, *χ^2^*(262) = 450.10, *p* < .001; CFI = .946; TLI = .936; RMSEA = .083, 90% CI [0.070, 0.096], with *χ^2^* = 231.52 for the feminine framed and *χ^2^* = 218.58 for the masculine framed leader role.

**Metric Measurement Invariance**

Metric invariance requires that factor loadings are equal across the groups. Therefore, the loading parameters of the measurement model were restricted to equality between the two framing of the leadership roles. The model fit and the model comparison with the previous model allowed conclusions regarding the metric measurement invariance across the two conditions, that is, whether the mean loadings are equal for the masculine and feminine framing of the leadership role. Model fit, *χ^2^*(276) = 458.12, *p* < .001; CFI = .947; TLI = .942; RMSEA = .079, 90% CI [0.066, 0.092], and the non-significant comparison with the previous model, Δ*χ^2^* = 8.02, Δ*df*= 14, *p* = .888, showed that metric measurement variance can be assumed.

**Scalar Measurement Invariance**

Scalar invariance requires that factor loadings and all intercepts are equal across the framings. Therefore, the intercepts of the manifest variables were also restricted to equality. The model fit and the model comparison with the previous model allowed conclusions regarding the assumption of scalar (“strong”) measurement invariance, that is, whether there was no differential item-functioning between the two conditions. Results showed that the model comparison was non-significant, Δ*χ^2^* = 3.88, Δ*df* = 14, *p* = .996, suggesting that strong scalar measurement invariance does hold. This assumption was further supported by the other criteria frequently used to establish strong measurement invariance, namely, that the more restrictive model should have a CFI/TLI that is less than 0.01 lower than the CFI/TLI of the less restrictive model, with ΔCFI = 0.003, ΔTLI = 0.006; ΔRMSEA = 0.004.

**PART 2: Mediation Model Additionally Including Physiological Stress Response**

An additional mediation model also included physiological stress response as a mediator, although this variable was not strongly correlated with the self-report and evaluation outcomes. As described in Table 3 in the main manuscript, we again used the area-under-the-curve with regard to the increase (AUC_i_; Pruessner et al., 2003) to map cortisol reactivity over time onto one variable. The model appears in Figure E1.

Results for this extended model showed an acceptable model fit, *χ^2^*(160) = 339.15, *p* < .001; CFI = .949; TLI = .939; RMSEA = .073, 90% CI [0.062, 0.084], accounting for 49.9% of the variance in self-evaluated interview success. Consistent with the correlations displayed in Table 3 in the main manuscript, the indirect effect via physiological stress response (i.e. AUC_i_) was nonsignificant (.003, 95% CI [-0.001, +0.021]). In fact, women reported lower perceived fit than men, *b* = -0.24, *p* < .001, which related to lower expected performance for the interview, *b*= 0.64, *p* < .001. Lower expected performance, however, was not related to physiological stress reactivity, *b* = 0.11, *p* = .235, and physiological stress reactivity also was not related to self-evaluated success, *b*= -0.08, *p* = .134.

**Supplement Figure E1**

*Structural Equation Model of the Effect of Participant Gender on Self-Evaluated Interview Success Through Perceived Fit, Expected Performance in Job Interview, Threat and Challenge Appraisal and Physiological Stress Reactivity (AUC_i_).*

Participant gender

-0.24***

-0.27**

0.09

-0.09

-0.31***

0.64****

0.03

*R^2^* = 49.9%

Expected
performance

Ratio threat vs. challenge appraisal

0.11

-0.06

Physiological stress reactivity (AUC_i_)

0.29***

-0.08

0.16 *

- 0.02

-0.15**

0.23**

*Note.* Standardized results are depicted. Significant paths are indicated by a solid line, and nonsignificant paths by a dashed line. Participant gender was coded man = 0, woman = 1. Ellipses represent latent variables, and rectangles represent observed variables. Physiological stress reactivity (i.e., Phase 1; assessed by t1, t2, and t3 cortisol measures) was mapped onto one variable to allow for inclusion in the mediation, following past work (e.g., Fay & Hüttges, 2017) we calculated the area-under-the-curve with regard to the increase (AUC_i_; Pruessner et al., 2003). * p *< .*05, ** p < .01, *** p < .001.

**PART 3: Simple Mediation Model Focusing on Self-Ascribed Agency**

A simple mediation model included self-ascribed agency as a mediator, namely, as a precursor of women’s, compared to men’s, greater lack-of-fit perceptions for leader roles. Results for this extended model showed a good model fit, *χ^2^*(25) = 39.21, *p* < .001; CFI = .985; TLI = .979; RMSEA = .052, 90% CI [0.014, 0.082], accounting for 11.4% of the variance in fit perceptions. The model appears in Figure E2.

The indirect effect of gender on perceived fit through self-ascribed agency was significant (-.10, 95% CI [-0.29, -0.01]). This indirect effect indicate that gender had a near-significant effect on agentic self-concept with women tending to describe themselves as having lower levels of agentic qualities than men (*β* = -0.15, *p* = .059), which related to lower perceived fit for leadership (*β* = 0.24, *p* = .002).

**Supplement Figure E2**

*Structural Equation Model of the Effect of Participant Gender on Perceived Fit Through Self-Ascribed Agency.*

Participant gender

.24**

-.15 †

*R^2^* = 11.4%

-.21**

*Note.* Standardized results are depicted. Significant paths are indicated by a solid line, and nonsignificant paths by a dashed line. Participant gender was coded man = 0, woman = 1. Ellipses represent latent variables, and rectangles represent observed variables.
† p < .06, * p < *.*05, ** p < .01, *** p < .001.

**References**

Milfont, T. L., & Fischer, R. (2010). Testing measurement invariance across groups: Applications in cross-cultural research. Inter*national Journal of Psychological Research, 3,* 111–130. https://doi.org/10.21500/20112084.857

Putnick, D. L., & Bornstein, M. H. (2016). Measurement invariance conventions and reporting: The state of the art and future directions for psychological research. *Developmental Review*, *41*, 71–90. https://doi.org/10.1016/j.dr.2016.06.004

Pruessner, J. C., Kirschbaum, C., Meinlschmid, G., & Hellhammer, D. H. (2003). Two formulas for computation of the area under the curve represent measures of total hormone concentration versus time-dependent change. *Psychoneuroendocrinology, 28(7),* 916–931. https://doi.org/10.1016/S0306-4530(02)00108-7

**Additional Tables & Figures**

**Table S1**

*Pearson Correlations for Women (Lower Triangle) and Men (Upper Triangle) Separately*

| Variables | 1 | 2 | 3 | 4 | 5 | 6 | 7 |
| --- | --- | --- | --- | --- | --- | --- | --- |

| 1. Framing of leader role | – | -.11 | -.13 | .04 | .12 | -.02 | .03 |
| --- | --- | --- | --- | --- | --- | --- | --- |
| 1. Perceived qualifications | -.24* | – | .67*** | -.31** | .16 | .52*** | .09 |
| 1. Expected performance | -.17 | .57*** | – | -.33*** | .22* | .51*** | .13 |
| 1. Appraisal of threat vs. challenge | .07 | -.16 | -.29** | – | -.02 | -.45*** | -.18 |
| 1. Cortisol AUCi Reactivity | .25** | -.04 | -.01 | .10 | – | -.02 | -.11 |
| 1. Self-evaluated interview success | -.09 | .44*** | .56*** | -.47*** | -.10 | – | .35*** |
| 1. Observer-evaluated interview success | .01 | .14 | .13 | -.26** | .01 | .34*** | – |

*Note*. Framing of leader role was coded feminine = 0, masculine = 1. Cortisol AUCi = Area under the curve with regard to increase (modeling reactivity or Phase 1 as assessed by t1, t2, and t3 cortisol measures).

* p < .05, ** p < .01, *** p < .001.

**Table S2**

*Means, Standard Deviations, for Appraisal of Threat and Appraisal of Challenge Separately, by Participant Gender and Role Framing*

|  | | |  |  | Appraisal of Threat | |  | Appraisal of Challenge | |
| --- | --- | --- | --- | --- | --- | --- | --- | --- | --- |
|  |  |  |  |  |  |  |  |  |  |
| Experimental design | | *N* |  |  | *M* | *SD* |  | *M* | *SD* |
| Women | Feminine | 64 |  |  | 3.37 | 1.39 |  | 5.05 | 1.09 |
|  | Masculine | 48 |  |  | 3.46 | 1.16 |  | 5.02 | 1.04 |
| Men | Feminine | 42 |  |  | 2.92 | 1.27 |  | 4.95 | 0.94 |
|  | Masculine | 55 |  |  | 2.91 | 1.15 |  | 4.90 | 1.01 |

*Note.* The scales for appraisal of threat and challenge ranged from (1) *strongly disagree* (7) *strongly agree.*

**Table S3**

*ANOVA Results for Appraisal of Threat and Appraisal of Challenge Separately, by Participant Gender and Role Framing*

| Source | *df* | *F* | *p* | η_p_^2^ |
| --- | --- | --- | --- | --- |
| Appraisal of Threat |  |  |  |  |
| Gender | 1 | 8.08 | .005 | <.001 |
| Framing | 1 | 0.06 | .812 | <.001 |
| Framing × Gender | 1 | 0.08 | .779 | <.001 |
| Error | 205 |  |  |  |
| Appraisal of Challenge |  |  |  |  |
| Gender | 1 | 0.62 | .431 | .003 |
| Framing | 1 | 0.10 | .754 | <.001 |
| Framing × Gender | 1 | 0.01 | .936 | <.001 |
| Error | 205 |  |  |  |

*Note*. 2 × 2 ANOVAs on appraisal of threat and challenge

**Figure S1**

*Laboratory Procedure With the Cessation of the Stressor*

t: -35 min

t: -50 min

t: -15 min

t: 0 min

t: +20 min

t: +40 min

Welcome &
 habituation phase

t: -5 min


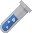

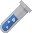

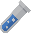

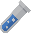


Job interview
(with self-presentation)


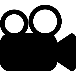


Framing of leader role


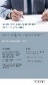


M


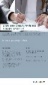


F

Personality questions


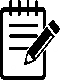


Perceived fit for role,
Expected performance


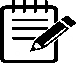


Threat vs. challenge appraisal, Self-evaluated interview success, Additional questions


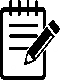


*Note*. The test tubes indicate the collection of salivary cortisol. The first sample (t1; baseline) was collected before participants learned about the leader role, the second (t2) after the job interview, the third (t3) and fourth (t4) followed 20 and 40 minutes after the interview.

**References**

Beilock, S. L., Rydell, R. J., & McConnell, A. R. (2007). Stereotype threat and working memory: Mechanisms, alleviation, and spillover. *Journal of Experimental Psychology: General*, *136*, 256. https://doi.org/10.1037/0096-3445.136.2.256

Bergeron, D. M., Block, C. J., & Echtenkamp, A. (2006). Disabling the able: Stereotype threat and women’s work performance. *Human Performance*, *19*, 133–158. https://doi.org/10.1207/s15327043hup1902_3

Coddington, R. D. (1972). The significance of life events as etiologic factors in the diseases of children—II a study of a normal population. *Journal of Psychosomatic Research*, *16*(3), 205–213. https://doi.org/10.1016/0022-3999(72)90045-1

Danner, D., Rammstedt, B., Bluemke, M., Lechner, C., Berres, S., Knopf, T., Soto, C. J., & John, O. P. (2019). Das Big Five Inventar 2: Validierung eines Persönlichkeitsinventars zur Erfassung von 5 Persönlichkeitsdomänen und 15 Facetten [The German Big Five Inventory 2]. *Diagnostica*, *65*, 121–132. https://doi.org/10.1026/0012-1924/a000218 ${

Fay, D., & Hüttges, A. (2017). Drawbacks of proactivity: Effects of daily proactivity on daily salivary cortisol and subjective well-being. *Journal of Occupational Health Psychology*, *22*, 429–442. https://doi.org/10.1037/ocp0000042

Ganzeboom, H. B. G., De Graaf, P. M., & Treiman, D. J. (1992). A standard international socio-economic index of occupational status. *Social Science Research*, *21*(1), 1–56. https://doi.org/10.1016/0049-089X(92)90017-B

International Labour Office. (2012). *International Standard Classification of Occupations (ISCO-08)*. International Labour Organization. http://www.ilo.org/public/english/bureau/stat/isco/isco08/

Löwe, B., Spitzer, R. L., Zipfel, S., & Herzog, W. (2002). *Gesundheitsfragebogen für Patienten (PHQ D)*. Pfizer.

Milfont, T. L., & Fischer, R. (2010). Testing measurement invariance across groups: Applications in cross-cultural research. *International Journal of Psychological Research*, *3*(1), 111–130. https://doi.org/10.21500/20112084.857

Putnick, D. L., & Bornstein, M. H. (2016). Measurement invariance conventions and reporting: The state of the art and future directions for psychological research. *Developmental Review*, *41*, 71–90. https://doi.org/10.1016/j.dr.2016.06.004

Rosenberg, M. (1965). *Society and the adolescent self-image*. Princeton University Press.

Soto, C. J., & John, O. P. (2017). The next Big Five Inventory (BFI-2): Developing and assessing a hierarchical model with 15 facets to enhance bandwidth, fidelity, and predictive power. *Journal of Personality and Social Psychology*, *113*, 117–143. https://doi.org/10.1037/pspp0000096

von Collani, G., & Herzberg, P. Y. (2003). Eine revidierte Fassung der deutschsprachigen Skala zum Selbstwertgefühl von Rosenberg [A revised version of the German adaptation of Rosenberg’s self-esteem scale]. *Zeitschrift Für Differentielle Und Diagnostische Psychologie*, *24*, 3–7. https://doi.org/10.1024//0170-1789.24.1.3
